# Translating knowledge for action against stroke – using 5-minute videos for stroke survivors and caregivers to improve post-stroke outcomes: study protocol for a randomized controlled trial (Movies4Stroke)

**DOI:** 10.1186/s13063-016-1175-x

**Published:** 2016-01-27

**Authors:** Ayeesha Kamran Kamal, Adeel Khoja, Bushra Usmani, Abdul Muqeet, Fabiha Zaidi, Masood Ahmed, Saadia Shakeel, Nabila Soomro, Ambreen Gowani, Nargis Asad, Asma Ahmed, Saleem Sayani, Iqbal Azam, Sarah Saleem

**Affiliations:** Section of Neurology, Department of Medicine, Aga Khan University, Stadium Road, 74800 Karachi, Pakistan; Stroke Service, the International Cerebrovascular Translational Clinical Research Training Program (Fogarty International Center, National Institutes of Health), Aga Khan University, Karachi, Pakistan; Aga Khan University, Karachi, Pakistan; Aga Khan Development Network, eHealth Resource Center, Karachi, Pakistan; Movies for Stroke Study, The International Cerebrovascular Translational Clinical Research Training Program (Fogarty International Center, National Institutes of Health) and Aga Khan University, Karachi, Pakistan; Section of Neurology, Department of Medicine, Aga Khan University and Director, Institute of Physical Medicine and Rehabilitation, Dow University of Health Sciences, Karachi, Pakistan; Stroke Service, Department of Medicine, Aga Khan University, Karachi, Pakistan; Department of Psychiatry, Aga Khan University, Karachi, Pakistan; Section of Diabetes, Endocrinology and Metabolism, Department of Medicine, Aga Khan University, Karachi, Pakistan; eHealth Resource Centre, Aga Khan Development Network, Karachi, Pakistan; Department of Community Health Sciences, Aga Khan University, Karachi, Pakistan

**Keywords:** Stroke, Educational intervention, Behavior change, Mobile health, Implementation, Information and communication technology, Prevention, Non-communicable disease, Low and middle income countries, Adherence

## Abstract

**Background:**

Two thirds of the global mortality of stroke is borne by low and middle income countries (LMICs). Pakistan is the world’s sixth most populous country with a stroke-vulnerable population and is without a single dedicated chronic care center. In order to provide evidence for a viable solution responsive to this health care gap, and leveraging the existing >70 % mobile phone density, we thought it rational to test the effectiveness of a mobile phone-based video intervention of short 5-minute movies to educate and support stroke survivors and their primary caregivers.

**Methods:**

Movies4Stroke will be a randomized control, outcome assessor blinded, parallel group, single center superiority trial. Participants with an acute stroke, medically stable, with mild to moderate disability and having a stable primary caregiver will be included. After obtaining informed consent the stroke survivor-caregiver dyad will be randomized. Intervention participants will have the movie program software installed in their phone, desktop, or Android device which will allow them to receive, view and repeat 5-minute videos on stroke-related topics at admission, discharge and first and third months after enrollment. The control arm will receive standard of care at an internationally accredited center with defined protocols. The primary outcome measure is medication adherence as ascertained by a locally validated Morisky Medication Adherence Scale and control of major risk factors such as blood pressure, blood sugar and blood cholesterol at 12 months post discharge. Secondary outcome measures are post-stroke complications and mortality, caregiver knowledge and change in functional outcomes after acute stroke at 1, 3, 6, 9 and 12 months. Movies4Stroke is designed to enroll 300 participant dyads after inflating 10 % to incorporate attrition and non-compliance and has been powered at 95 % to detect a 15 % difference between intervention and usual care arm. Analysis will be done by the intention-to-treat principle.

**Discussion:**

Movies4Stroke is a randomized trial testing an application aimed at supporting caregivers and stroke survivors in a LMIC with no rehabilitation or chronic support systems.

**Trial registration:**

NCT02202330 (28 January 2015)

**Electronic supplementary material:**

The online version of this article (doi:10.1186/s13063-016-1175-x) contains supplementary material, which is available to authorized users.

## Background

The World Health Organization (WHO) defines stroke as “a focal (or at times global) neurological impairment of sudden onset, and lasting more than 24 hours (or leading to death) and of presumed vascular origin” [1]. Two thirds, i.e., 36 million of the estimated 56 million deaths worldwide are attributed to non-communicable diseases (NCD), primarily stroke. Over 80 % of strokes now occur in LMICs [2], with 15 million strokes occurring annually worldwide, leaving five million dead, and five million permanently disabled. One person suffers a stroke every 45 seconds; one person dies of a stroke every 4 minutes [3]. Globally, stroke accounts for 46.6 million disability adjusted life-years (DALYs) [4].

NCD and stroke are becoming an important issue in Pakistan as the country undergoes demographic transition [5]. Pakistan, the sixth most populous nation in the world, has a distinctively stroke-prone population. Today, about one in four adult Pakistanis has hypertension and/or diabetes, heart disease, or a stroke equivalent [6]. Yet, early detection and risk reduction would have prevented stroke in 80–90 % of victims [7]. A local study conducted on one ethnicity (Pashtoon), reported a prevalence of 4.8 %, which would translate into four million persons in a country of 180 million [6]. In another hospital-based study, 65 % of adult Pakistani stroke survivors reported at least one post-stroke complication and a 12 % mortality in the first year post discharge [8].

This high-risk population is also highly underserved. Health systems are acutely responsive and are not equipped to respond to chronic diseases. There is not a single inpatient comprehensive rehabilitation system. Since the recent democratic reforms and 18th Constitutional Amendment, an opportunity has arisen for health reform, where devolution has resulted in provincial autonomy for health [9]. Thus, evidence for a systematized, replicable, scalable and effective response to chronic disorders is much more likely to be adapted.

In 80 % of stroke victims, further events may be prevented by altering lifestyle risk factors, and increasing adherence to medications to control hypertension, diabetes and hyperlipidemia [10–12]. However, there exists an implementation gap to adopt widely recognized beneficial medicines and lifestyle changes after stroke [6].

In this study, we will assess the effect of video-based intervention on primary stroke survivors and their caregiver dyads on improving adherence to stroke preventive medications and control of three major risk factors at 6 and 12 months in a Pakistani population. Our rationale is to leverage the existing mobile phone infrastructure and use IT to deliver post-stroke education to dyads returning to the community and thereby improve post-stroke outcomes. We hypothesize that there would be a difference of at least 15 % in adherence to stroke preventive medications and control of three major risk factors at 6 and 12 months among stroke survivors who are given video-based education as compare to those who are given usual care.

### Study question

Does video-based intervention given to stroke survivors (with mild to moderate disability) and their caregivers at the time of enrollment into the study, at discharge and sustained during follow-up, improve adherence to stroke preventive medications and control of three major risk factors (diabetes, hypertension and dyslipidemia) at 6 and 12 months after first ever stroke?

### Primary objective

To determine the effect of video-based education intervention provided to patient and caregiver dyads after first ever stroke on:

- Proportion of participants adhering to medications prescribed (definition of adherence is the use of prescribed medications on at least 5 days a week and measured by the Morisky Medication Adherence Scale)
- Proportion of participants achieving control of blood pressure (control is defined as BP <125/85), blood sugar (glycosylated hemoglobin (HbA1c) <7 %) and blood cholesterol (low-density lipoprotein cholesterol (LDL) <100 mg/dl)

### Secondary objective

To determine the effect of video-based education intervention provided to patient and caregiver dyads after first ever stroke on:

- Stroke-related knowledge assessment among caregivers regarding risk factors, emergency preparedness, stroke rehabilitation and medications
- Readmission to hospital with any of the stroke-related complications – urinary tract infection, pneumonia, and deep venous thrombosis, among stroke survivors admitted to the AKUH Stroke Unit, Karachi
- Stroke-related mortality among stroke survivors with mild to moderate disability admitted to the AKUH Stroke Unit, Karachi
- Change in functional outcome (severity and disability after stroke) from baseline among stroke survivors admitted to the AKUH Stroke Unit, Karachi
- Level of satisfaction with the intervention and acceptability of mobile health (mHealth) innovations by stroke survivor and caregiver dyads

## Methods

### Study design

**Fig. 1:**
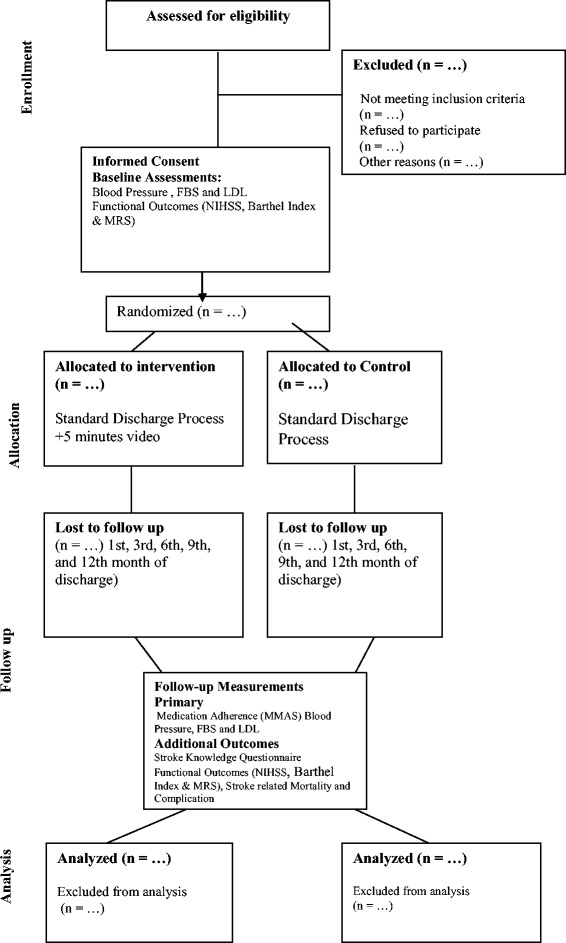
Study flow chart

This will be a randomized controlled, outcome assessor blinded, parallel group, single center superiority trial in which participants with first ever stroke (both ischemic and hemorrhagic) will be randomized within 48 hours of their stroke to either the video-based education intervention group or the control group [13]. The video-based education intervention group will have health education delivered through short videos which will be shown to the participants and their caregivers at the time of admission, before discharge, and follow-up at first and third month post discharge. The control group will have standard care including pre-discharge education and counseling according to defined protocol. All participants enrolled in this video education intervention group and control group will be followed for 12 months post discharge for assessment of end-points (Refer to Fig. 1).

### Study setting

The trial will be conducted in the Stroke Unit, Neurology ward at Aga Khan University Hospital (AKUH) Karachi, Pakistan. AKUH is an internationally recognized tertiary care institution certified by Joint Commission International Accreditation (JCIA) that caters to the needs of large multi-ethnic urban population of 18 million.

Due to the high variability of stroke care being delivered in different centers of Karachi and the need to evaluate a complex multimodal intervention against a uniform standard of care, we chose to limit our study to a single center. Usual care at AKUH involves a proper stroke pathway which includes a standard protocol being followed by a neuro-physician, a stroke nurse, a dietitian and a physiotherapist in accordance with institutional guidelines (please refer to Standard Care for Stroke Survivors for details).

### Population

The following criteria will be used while recruiting participants and caregivers throughout the study:

### Stroke survivor

Any adult aged 18 years of age or older, having experienced first ever stroke (ischemic or hemorrhagic) within the past 6 weeks, with mild to moderate disability. Stroke is clinically stable and the stroke survivor is returning to the community for chronic care.

### Caregiver

An adult aged 18 years of age or older who is present 24 hours a day with the stroke survivor and provides the overall day-to-day care of the stroke survivor, including acute emergency care, appointments, and/or follow-up visits.

### Android mobile

Any portable telecommunication device based on the Android Operating System, in the possession of either the stroke survivor or the caregiver, which is compatible with our Movies4Stroke Application.

### Eligibility criteria

#### Inclusion criteria

- Adult men and women, 18 years of age or older
- Resident of Karachi and planning to live in Karachi till the follow-up period
- Able to understand Urdu (language of the videos)
- Admitted with first ever stroke (ischemic or hemorrhagic stroke)
- Modified Rankin score (mRS) ≤4 ( mild to moderate stroke)
- Have at least one vascular risk factor that requires medical intervention
- Consenting to participate in the study and follow-up, both stroke survivor and caregiver
- Have a stable surrogate caregiver at home who is responsible for appointments, follow-ups, overall care, e.g., wife, daughters, daughter- in- law, husband
- Stroke is medically stable and participant is likely to return to the community after the in- hospital stay (thus, actively treated strokes, e.g., decompressive surgeries, carotid endarterectomy (CEA), in-hospital sepsis, ventilator complications) that essentially preclude return to the community settings will not be offered this chronic care support study

### Exclusion criteria

- Serious aphasia, visual hemi-neglect, short-term memory loss in the stroke survivor precluding understanding, visualization or retention of the video material (will be measured through the NIH Stroke Scale performed by trained physicians; those having a score of greater than 4 due to aphasia alone will be excluded)
- Serious aphasia, visual hemi-neglect, short-term memory loss, dementia in the caregiver precluding understanding, visualization or retention of the video material (will be measured through the NIH Stroke Scale performed by trained physicians; those having a score of greater than 4 due to aphasia alone will be excluded, dementia status will be assessed by the Mini-Mental State Examination)
- Iatrogenic stroke, i.e., stroke due to non-atherosclerotic vascular disease and rare causes, e.g., carotid dissections, gunshot wounds to the neck, post coronary artery bypass grafting (CABG)
- Stroke survivor/Caregiver dyad continue post-stroke care in a nursing assisted, professional or hospital setting and do not return to the community after discharge
- Serious concurrent medical illnesses, like cancer, renal failure, acute liver disease in past 6 months (that precludes the use of statins), chronic liver disease that excludes the use of stroke preventive medications, or require non-standardized therapy
- Use of any off-label, non-guideline medications which, due to stroke survivors’ unique co-morbidities, interfere with medication compliance to antihypertensives, statins, antiplatelet agents and diabetes control

### Identification and enrollment of the study participants

**Fig. 2:**
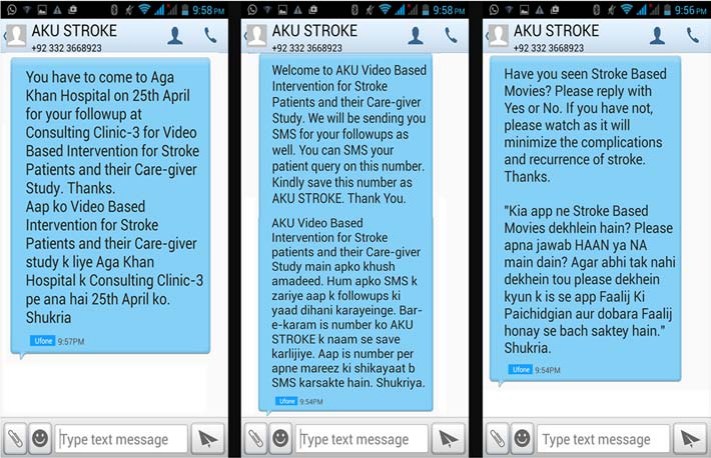
Template for screenshot of short messaging service (SMS)

Data collectors will screen the participants coming to AKUH Stroke Unit, Neurology ward, Karachi. Data collectors will fill out the eligibility form and, if the participants are found eligible for the study, they will be officially invited to participate in the trial. Informed consent will be obtained from those participants who voluntarily wish to be in our study. The study participants and their caregivers will be thoroughly counseled about the nature of the study and required follow-up. It will also be explained to study participants that the selection in the study group is random. Stroke survivors will then undergo a detailed interview regarding the socio-demographic, clinical and functional assessment at baseline. Then, the participants will undergo randomization to either the intervention group or standard care group. After allocation assignment, the research supervisor will thoroughly explain the details of the software program and its installation and then proceed with the delivery of the first set of 5- minute videos. Any questions pertaining to the use and the comprehension of the videos and software will be answered. Videos will be shown at the time of enrollment into the study, at discharge from the hospital, and follow-up at first and third months post discharge and a memory card containing the Stroke4Movie Application will be installed and programmed to deliver scheduled videos on the participant’s Android phone. In case of the participant being allocated to the usual care group they will be informed about the details regarding discharge and the follow-up appointments at the clinic (Refer to Fig. 2) (see Additional files 1, 2 and 3).

### Sampling technique

A purposive sampling technique would be applied to select adult stroke survivors coming to the Stroke Unit, Neurology ward, AKUH, Karachi after the assessment of the eligibility criteria and informed consent. The purposive sampling technique is defined as the sample based on researchers’ established criteria for eligibility. Access to daily admission lists of stroke patients to all areas of the hospital will serve as the reference sampling frame for this exercise.

### Randomization process

A computer-generated random number list will be used to randomize subjects to the control arm or the intervention arm. The randomization center will be at a secure computer in the Clinical Trials Unit (CTU) and the random number will be generated by the staff of the CTU who are not involved in recruitment and/or outcome ascertainment.

### Allocation concealment

Randomization sequence will not in any way be predictable as it will be centralized. No-one from the research team will have access to the randomization sequence, block size, randomization envelopes or code. Envelopes will be sealed, opaque and it will be impossible to view the code even if it is held against bright sunlight.

### Sample size calculation

The study is designed to enroll 300 participant dyads (150 participants and 150 caregivers in each group – intervention versus standard care) after inflating 10 % to incorporate attrition and non-compliance. The sample size was calculated keeping in mind the two primary end-points of medication adherence and risk factor control; the primary end-point that gave the highest sample size was kept as the final sample size. This number of participants would provide the study with the ability to detect a 15 % difference among groups with a power of at least 95 %. The proportion of participants achieving control of three modifiable risk factors (blood pressure, blood cholesterol and blood sugar) was assumed to be 0.50 in the treatment group and 0.350 in the control group. The test statistics used was a two-sided *Z* test. The sample size was calculated using NCSS PASS (version 2008).

### Statistical analysis plan

Analysis will be performed on the basis of initial treatment allocation; that is the intention-to- treat (ITT) principle.

### Checking for normality and descriptive analysis

Histograms will be graphed to check the distribution of all the continuous variables in the dataset. For descriptive analysis, mean with standard deviation will be reported for symmetrically distributed continuous variables while median with interquartile range will be reported for not symmetrically distributed continuous variables. For categorical data, frequency with percentages will be reported.

### Univariate analysis

Scale examination of all the continuous variables will be carried out. Some of the continuous variables will be transformed into categories where needed. We might need to collapse categories for categorical variables if we have a sparse data problem. Multiple Linear Regression (MLR) will be applied at the univariate analysis level. Each variable will be regressed independently with the primary end-point. Their assumptions will be checked and their significance will be reported. At univariate analysis level, a variable will be considered to be biologically significant as well as statistically significant if the *p* value is less than or equal to 0.10. Crude beta-coefficient with 95 % confidence interval will also be reported.

### Multivariable analysis

A variable having a *p* value of less than or equal to 0.05 will be considered to be clinically significant as well as statistically significant at the multivariable analysis level. All variables that are considered to be significant at the univariate analysis level will need to run through a stepwise model building approach (manual) to obtain a parsimonious model. Multiple linear regression (MLR) will be used to predict the final model. Adjusted beta-coefficients with their appropriate 95 % confidence interval will be reported for the final model. The final model will be assessed for interaction between biologically plausible variables as well as for confounding after checking for interaction; beta-change (15 %) will be considered significant for confounding to be reported. Assessment of multicollinearity between two independent variables will be carried out using appropriate statistical tests. A correlation coefficient of more than 0.80 will be treated as significant for multicollinearity. The model will be assessed for the presence of outliers and influential observations and robust standard errors will be reported if influential observations are found.

### Secondary end-points

To analyze the secondary outcome, caregivers’ mean score of knowledge regarding stroke risk factors, stroke rehabilitation and medications at each follow-up visit between the two groups will be calculated through Student’s *T* test if the outcome is symmetrically distributed or the Mann-Whitney *U* test if the outcome is not symmetrically distributed.

The proportion of participants with respect to readmission to hospital with any of the stroke-related complications between the two groups will be calculated either by Pearson’s chi-square test or Fischer’s exact test (if the expected cell count is less than 5).

The proportion of participants with respect to stroke-related mortality between the two groups will be calculated either by Pearson’s chi-square test or Fischer’s exact test (if the expected cell count is less than 5).

The change in mean score of National Institutes of Health Stroke Scale (NIHSS), Barthel Index and mRS from baseline at each follow-up visit between the two groups, will be calculated through Student’s *T* test if the outcome is symmetrically distributed or the Mann-Whitney *U* test if the outcome is not symmetrically distributed.

The change in mean scores for level of satisfaction with the intervention and acceptability of mHealth innovation at the third month follow-up visit post discharge between the two groups will be calculated through Student’s *T* test if the outcome is symmetrically distributed or the Mann-Whitney *U* test if the outcome is not symmetrically distributed.

### Outcome assessment

The outcome assessment will be at 1, 3, 6, 9 and 12 months post discharge. Assessors will not be allowed to view the cell phone of the participant and they will be instructed not to ask participants the following question: “Did you watch any videos on stroke?” They will be trained to accept random quality cross checks. A random cross check will be done of assessor quality by asking 30 % of the participants if they were asked the above question on assessments. Since our outcome assessment will be blinded and, if an unblinding/protocol violation has occurred at the assessor level, the participant will be removed from the final analysis to avoid assessor bias that may shift the outcome in favor of the intervention.

At an assessment visit:

1. A detailed questionnaire will be filled out which will collect information on:
  Medication adherence
  History of hospitalizations since discharge, with any of the stroke complications and associated mortality
  Knowledge level of stroke survivor/caregiver
2. Stroke survivors will have blood pressure measurement, two separate readings at least 10 minutes apart, at resting and in sitting positions
3. Participants will be asked to give blood samples for serum cholesterol, fasting blood sugar and HbA1c (if diabetic) at 6 and 12 months post discharge

### Primary study outcomes

All participants will be assessed for outcomes at 1, 3, 6, 9 and 12 months post discharge. The two arms will be compared with respect to:

1. Proportion of participants adhering to medications prescribed (definition of adherence – use of prescribed medications on at least 5 days a week and measured by the locally validated Morisky Medication Adherence Scale [14]
2. Proportion of participants achieving control of blood pressure (control is defined as BP <125/85), blood sugar (HbA1c <7 %) and blood cholesterol (LDL <100 mg/dl) [14]

### Secondary study outcomes

1. Change in knowledge of caregivers concerning stroke risk factors, stroke rehabilitation and medications (a knowledge questionnaire will be administered at 1, 3, 6, 9 and 12 months post discharge and change in score will be ascertained)
2. Readmission to hospital with any of the stroke-related complications – urinary tract infection, pneumonia, and deep venous thrombosis (this information will be elicited at the follow-up time, based on recall. All participants will be asked to bring documents related to any hospital admissions in the period following discharge)
3. Stroke-related mortality will be assessed through a Verbal Autopsy Scale adopted from the WHO [15]
4. Change in functional outcome (severity and disability after stroke) from baseline among stroke survivors will be assessed through validated tools (NIHSS, Barthel Index and mRS) [16]
5. Level of satisfaction with the intervention and acceptability of mHealth innovation such as videos on selected topics among stroke survivors and caregiver dyads to improve clinical outcomes. This will be done on a self-designed tool which ascertains the constructive and unpleasant attributes of using this mHealth technology (please refer to Form C, Data Collection Form for Stroke Survivors– see Additional file 1). This self-designed tool will be administered by a research supervisor who will be unblinded to the randomization group. This tool has design attributes based on Rogers’ Diffusion of Innovation Theory for ease, adoption, repeatability, and adaptability [17].

### Intervention

Intervention consists of:-

- 5-minute videos, on various stroke-related topics/themes delivered at the time of enrollment into the study, before discharge from the hospital, at first and third month follow-up post discharge
- Session 1 will be delivered at the time of enrollment into the study and will cover themes on information for caregivers including recognition of stroke, and different skills will be taught to the caregivers including swallowing exercises, different rehabilitation exercises and naso-gastric tube feeding
- Session 2 will be delivered at the time of discharge from the hospital and will include videos on emergency preparedness – cardiopulmonary resuscitation, seizures, heart attack, hypoglycemia and recurrent stroke
- Session 3 will be delivered at first month follow-up post discharge and will include videos on frequently used medications by stroke survivors, such as anticoagulant, antihypertensive and lipid-lowering drugs
- Session 4 will be delivered at third month follow-up post discharge and will include videos on secondary stroke prevention (recurrent attack) – exercise, physical activity, recognition of depression, diet modification and accurate measurement of blood pressure and blood sugar
- Please refer to Additional file 4 (Video Thematic Intervention Chart) which demonstrates the core messages that will be delivered in the videos
- Discussion and question and answers after viewing each set of videos to ensure that the core message has been understood and there are no lacunae in understanding the message imparted by the videos, which may be re-wound and re-run for those who could not understand a particular message
- Movies4Stroke Application Installation in a memory card – a memory card containing the movies application will be installed in the participants’ cell phones in the intervention group to ensure that the videos can be replayed at home to refresh the memory of various details which may not have been captured in the mandatory viewing session that captures the usage data

### Compliance during the administration of intervention

The study officer, after receiving randomization to the intervention group will perform the following functions:

- Ensure that there is space and time for both caregiver and stroke survivor to feasibly watch the videos and that both are receptive
- Ensure that there is no contamination by delivering the video in a non-public area – participant’s room if private or semi-private, counseling room if a general ward patient
- Ensure that the sound quality is at a level that is clear to both stroke survivor and caregiver and that both are receptive
- Ensure that the caregiver sees all the videos in the tablet at least once as administered by the unblinded study officer
- Check if the videos can be replayed on the dyad’s home Android systems and that there are no technical issues

### Video translation and adaptation

The science in the videos is based on class I A evidence [18, 19]. However, the language of the instructions and the setting will be tailored according to culturally accepted norms [20]. Specifically, we want local resonance and acceptability [21]. Videos prior to being launched in the field will be shown to laypersons, stroke survivors and stroke caregivers to obtain feedback. Since this is a digital media, editing the same is feasible. Those who will assess the movie content in the pre-testing phase of the intervention will not be recruited in this implementation study.

The 5-minute idea is to hold attention, offer repeatability and engender self-efficacy [20, 22]. There should be sense of empowerment with manageable information. This is very important for cognitive, literacy and numeracy impaired populations [23]. For future scalability, digital media that last for 8–10 minutes can take excessive space and time downloading which can lead to user frustration and failure [24, 25]. Since these videos will be a resource, they should tend to be user friendly across platforms and systems, and time compression would achieve that goal. A similar concept has also been utilized in medical education internationally [26].

### Measuring adherence to video intervention

**Fig. 3:**
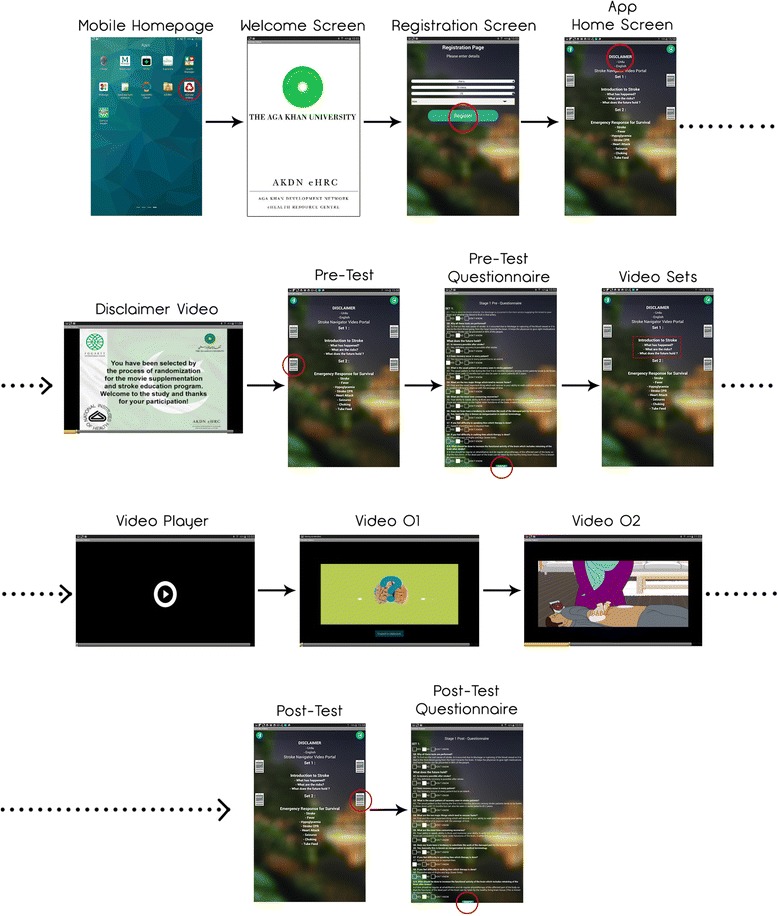
User interface snapshots of the movie software

To ensure adherence to intervention in the video group, at least one viewing will be delivered in the hospital or the CTU through active involvement of stroke survivors and their caregivers. In order to ensure that each important learning point is made clear in the first session, the key pre-test and post-test knowledge questions, which will be embedded in the movie application software for each set of movies, will form a template for testing and discussion. Throughout the videos, both stroke survivors and their caregivers will be encouraged to ask questions and clear their queries with the respective team members, and questions and answers accompany each video to provide a platform for discussion and comprehension. Since learning is enhanced by repetition, we will enable repeat viewing with a phone card memory application that contains the relevant videos [27]. Thus, those who are too stressed to learn in the first place, or who are older adult learners, will have the ability to review the videos in addition to the mandatory session. The Movies4Stroke Application Software (Refer to Fig. 3) will have the ability to monitor video usage and views by the user. This will help us in calculating the ‘sticky time’ (time spent using and interacting with the movie application software), the rationale being that we will be able to track actual usage of our intervention in the video arm in real time.

### Standard of care for stroke survivors (control group)

Stroke survivors will be given instructions before discharge regarding diet, need for rehabilitation, possible complications, and medication use and information booklets will also be handed out. The information will be imparted by a multidisciplinary team consisting of a neuro-physician, a stroke nurse, a dietitian and a physiotherapist. There will also be verbal instructions given to the stroke survivors and their caregivers. On the day of discharge, or 24 hours prior to discharge, a discharge coordinator will give the details about the skills learnt and will ensure that the medical, social, and rehabilitation requirements are in place prior to leaving hospital. All acute stroke survivors will be given follow-up appointments at the clinic. A detailed written discharge summary will be given out to the caregiver detailing all aspects of care, follow-up, medications, test results and serious alerts. This standard of care will be followed for all participants including those who receive video-based intervention.

### Operational plan

#### Pre-field activities

##### Hiring and training of staff

Four experienced research associates will be recruited for a total duration of 12 months. One research supervisor will be recruited full-time for a total duration of 15 months. The research supervisor should at least possess an MBBS degree with relevant experience in clinical neurology. All the members in the team including the research supervisor will be given formal training in interviewing (data collection) for at least 2 weeks.

Research training will emphasize on the following areas:

- Introduction of the Study Project and the concerned team members
- Administration of the questionnaire and its completeness including the quality control procedures and compliance during the administration of the intervention
- Contact information and follow-up procedures
- Emphasis on communication skills and how the data collectors should communicate with the study participants and their caregivers
- Procedure pertaining to informed consent
- Introduction to the video intervention and technical aspects of the software

### Pre-testing of the questionnaire

Pre-testing will be done on 20 % of the total sample size, that is 60 participant dyads, and those participants will be excluded from the final analysis. Questions that will require some changes due to phrasing and improper translation will be amended after pre-testing.

### Instructions for follow-up

Each stroke survivor caregiver dyad will be required to follow up at 1, 3, 6, 9 and 12 months post discharge in the neurology clinic for outcome ascertainment. Stroke survivors and their caregivers will be given a handout which will have instructions and basic information about their follow-up visits. Follow-up visit explanations will be given verbally to caregivers together with emphasizing the importance of their patient’s follow-up visit. They will be asked to contact the study team through our Stroke Helpline for any queries that arise in the interim period.

### Follow-up visits in the neurology clinic

Follow-up visits at 1, 3, 6, 9 and 12 months will be organized in the neurology outpatient clinic to assess outcome. In order to maximize our follow-up, participants will be sent short messaging service (SMS) reminders through our Stroke Helpline a day prior to the visit and transport will be arranged to facilitate the visit. If a stroke survivor along with their caregiver is not able to report for follow-up on the exact date, a grace period of ±14 days will be permitted to ensure capturing maximum follow-up. Those participant dyads that cannot appear for the follow-up in person will be contacted via phone or through establishing a follow-up visit appointment in synchrony with other clinical visits, physiotherapy sessions and laboratory investigations.

### Data management

#### Data management plan

A data entry officer will work in conjunction with the principal investigator (PI). The data entry officer will be given a formal training session on how to read the filled data forms and then enter it in the database. A codebook that contains specific number codes for each item of the questionnaire used in the data collection forms will be introduced to the data entry officer. The PI will request the data entry officer to enter the data for the five study participants in front of the PI so that any mistakes or discrepancies can be immediately rectified. A similar quality cross check will be performed during the double data entry process.

### Data editing

Data editing will ensure a complete data file for each participant. This will be done to check for any missing, incomplete or incorrect information provided at the time of interview by a stroke survivor or their caregiver. The data collectors will edit the filled questionnaire on a daily basis. Editing will be performed in two stages in order to avoid missing or inappropriate information. The field editing will be done by interviewers, just after the data collection process so that missing information can be obtained and any inappropriate information can be corrected. In case of missing or inappropriate information, the PI will contact the participant on their cell/home phone number for correction. Any missing information or discrepancies will be clarified immediately; every effort will be made to complete the information. The data will be double checked by a research supervisor and the PI on a weekly basis.

### Data entry

The data entry program will be created in EpiData version 3.1. Data will be double entered by two independent data entry operators. The two datasets will then be compared for the consistency of data and for missing values. Correction will be made by referring to a particular question.

### Data storage

Designated computers that are based at the CTU will store the data. Only research staff will be authorized entry into the system computers. All source documents will be maintained in locked files in locked rooms in accordance with patient privacy and confidentiality. After the study period is over, data will be stored for another 5 years as per institutional guidelines.

### Ethical considerations

Our study was approved by the Ethical Review Committee of Aga Khan University (AKU), Karachi (ERC # 2890-Med-ERC-14). Principles of autonomy, anonymity, confidentiality and equity will be followed throughout the study. Importantly, all videos will be viewed privately. No video or phone will have any icon that suggests that the person has a disease. No phone information of individuals will be made public even within the team; only coordinators or those with direct patient contact will have access to this information.

All videos developed will be ultimately shared with all participants as well as within the community so as to maximize the benefit to people of the educational tool.

Any modifications or changes in the protocol shall be communicated to the Ethical Review Committee and implemented after approval.

### Informed consent procedures

All participants and their caregivers will provide informed consent. This will be sought via trained research staff who will tell them exactly what time commitments are being sought from them. We will document oral and written consent to participation. Prior to testing we will train all research personnel about informed consent procedures. All participants and their caregivers may choose to withdraw at any time (see Additional file 5).

Informed consent will be taken separately from both the stroke survivors and their caregivers who voluntarily agree to participate in the study.

### Dissemination

Dissemination through publications, research seminars, conferences and mass media will be done. Free upload and sharing of video education will be available at the AKU website after due clearances are made with IT, University Research Council (URC), the Research Office and media group of AKUH.

## Discussion

The overarching goal of this clinical trial is to test the power of IT-based education using Android handsets as vehicles for teaching post-stroke survival skills to stroke survivors and caregiver dyads in a low and middle income country (LMIC) setting [28] (refer to Additional file 6 for SPIRIT Checklist). Previous studies that have used education to assist stroke survivors in communities are mainly based in developed countries (see Additional file 7).

A randomized controlled, single center, clinical trial assessed the effect of training of caregivers of stroke participants in a stroke rehabilitation unit on discharge, at 3 and at 12 months [28]. This study showed a significant difference between the trained group and conventional caregivers with respect to quality of life and mood outcomes, and burden of care, as well as cost-effectiveness. Another multicenter study that enabled caregivers during inpatient hospitalization, recommended more follow-up training after discharge due to lack of sustained effect [29, 30]. A Cochrane Review based on caregiver enablement via non- pharmacological interventions listing eight studies with 1007 participants, with heterogeneous follow-ups, interventions and settings, concluded that more work was needed in this area due to heterogeneity of effect [31]. None of these interventions took into consideration resource-poor, health-illiterate populations, fragmented health systems, attention to scalability, or adaptation to local needs, or used digital media.

The health care system in Pakistan represents a unique challenge. Like other LMICs with rapid demographic transition, there is now a double burden of maternal and childhood illnesses along with a significant additional NCD burden [32]. However, the health systems are not prepared for this challenge. There is not a single inpatient rehabilitation center for stroke survivors and the majority of care is provided directly by caregivers [5, 33, 34].

While there is a relative lag in development of such centers, we plan to test and leverage our existing mobile infrastructure as a means of providing continuous care. Based on the current statistics by the Pakistan Telecommunication Authority, mobile phone users in Pakistan were recorded to number greater than 137 million and total cellular density had reached 77 % by 2014 [35]. In addition, mobile brands have introduced cheap Android Smart phones that are rapidly becoming mainstream and are expected to take over the larger market share, with the cheapest Smart phone starting from around US$6 [25]. Thus, we developed this interactive Android application.

mHealth applications have tremendous potential for chronic disease support. However, these software applications have not been tested in rigorous trial designs with clinically meaningful outcomes [36–38, 39].

The strength of this study is the use of a rigorous randomized controlled trial (RCT) design, measurement of intervention usage and fidelity, intervention delivery that is present both within the hospital and at follow-up, evaluation of clinically important outcomes and ITT analysis [40].

The limitations include the following: selection bias: this is a single center study chosen because of the fact that this site provides a standard of care that is algorithmic and replicable; thus, any results may be attributed to the intervention. In this study we may report its efficacy, but the performance in different sites may vary; contamination bias: in an educational intervention, care will have to be taken to avoid contamination of the non-intervention arm with the intervention. To ensure this, videos will be shown in a separate room, and not at the bedside. Given the fact that families do share information, contamination is possible; however, most stroke participants are on different schedules for visits, rehabilitation times, etc. and so we expect less contamination than in areas where a lot of time is spent together by families; attrition bias: many participants may drop out due to the longitudinal nature of the study. To counteract this, trained health care providers will try to follow the subjects closely through regular phone calls and build good rapport with them. A Stroke Helpline number will also be provided.

If this initial study shows promising results, we will plan a larger study where interactive chronic disease support software will be evaluated for reduction in mortality and disability after acute stroke for families living in LMIC settings.

### Trial status

Ongoing

## Additional files

Additional file 1:
**Data Collection Form (DCF) for Stroke Survivors.** (DOC 771 kb) Additional file 2:
**Data Collection Form (DCF) for Caregiver.** (DOC 209 kb) Additional file 3:
**Participant timeline.** (DOCX 48 kb) Additional file 4:
**Thematic Intervention Chart.** (DOCX 17 kb) Additional file 5:
**Consent Form.** (DOCX 20 kb) Additional file 6:
**SPIRIT checklist.** (DOC 122 kb) Additional file 7:
**Review of previous studies.** (DOCX 31 kb)

## Abbreviations

AKDN: Aga Khan Development Network
AKU: Aga Khan University
AKUH: Aga Khan University Hospital
CAE: Carotid artery endarterectomy
CTU: Clinical Trials Unit
e-HRC: E-Health Resource Center
JCIA: Joint Commission International Accreditation
LMIC: Low and middle income country
mHealth: Mobile health
MMAS: Morisky Medication Adherence Scale
NCD: Non-communicable diseases
PI: Principal investigator
RCT: Randomized control trial
SMS: Short messaging service
WHO: World Health Organization
