# Supplementary material for: Translating knowledge for action against stroke – using 5-minute videos for stroke survivors and caregivers to improve post-stroke outcomes: study protocol for a randomized controlled trial (Movies4Stroke)
Source: Trials. 2016 Jan 27;17:52. doi: 10.1186/s13063-016-1175-x (PMC4728820; doi:10.1186/s13063-016-1175-x)
Supplement: Additional file 1: — Data Collection Form (DCF) for Stroke Survivors. (DOC 771 kb) [file 13063_2016_1175_MOESM1_ESM.doc]

**Appendix**

**ELIGIBILITY FORM**

**IDENTIFICATION DATA:**

1. Serial Number for eligibility :- _______________________
2. Medical Record No. :- _________________________
3. Name of the patient:- _______________________________
4. Mobile Number of the patient :- _______________________
5. Address of the patient:- _________________________________________________
6. Name of the Care-giver:- ____________________________
7. Mobile Number of the Care-giver:- ____________________
8. Address of the Care-giver:- ______________________________________________

**PATIENTS FILE REVIEW:**

Date of Medical File Review: ___________________

| **S.No** | **Eligibility Criteria** | **Yes** | **No** |
| --- | --- | --- | --- |
| 1 | Age >18 yrs. |  |  |
| 2 | Admitted with first ever stroke (acute stroke) |  |  |
| 3 | Able to understand Urdu (languages of the video) |  |  |
| 4 | Have a stable surrogate care-giver |  |  |
| 5 | Modified Rankin Score < 4 (mild to moderate stroke) |  |  |
| 6 | No intention to travel for next 12 months |  |  |
| 7 | Permanent resident of Karachi |  |  |
| 8 | No history of short term memory loss or any visual or hearing loss in the patient and caregiver that interferes with the understanding of the videos |  |  |
| 9 | No history of serious concurrent medical illnesses, like cancer, renal failure , chronic liver disease or acute liver disease in the past 6 months |  |  |
| 10 | Those patients not to be included having non- atherosclerotic vascular stroke or stroke from rare causes for e.g. gunshot to neck, carotid dissection and post CABG ,etc. |  |  |
| 11 | No history of any unique co morbid that interfere with patient’s medication compliance e.g. a liver failure patient that can’t take statins or a surgery is planned so aspirin needs to be stopped. |  |  |

All criteria should be answered in YES to be eligible for participation in the study (both patient along with caregiver).

**This patient is eligible to participate in the Video-Based Intervention for stroke patients and caregivers**

**YES NO**

If the patient and care-giver are eligible but refuses to participate please state the reason for

Non-participation: ______________________________________

**MOVIES FOR STROKE PATIENTS/CAREGIVER DYAD**

**PHONE INFRA STRUCTURE DATA**

1. **BRAND NAME OF THE CELL PHONE: _________________________**
2. **TYPE OF OPERATING SYSTEM IN THE CELL PHONE:**

**i) ANDROID**

**ii) WINDOWS**

**iii) IOS**

1. **DOES ANY ONE AT HOME HAVE AN ANDROID CELLULAR PHONE?**
2. **YES**
3. **NO**
4. **DO YOU HAVE A DESKTOP PC AT HOME?**
5. **YES**
6. **NO**

**Data Collection Form For Patients**

Study time point: T0□ T1□ T2□ T3*□ T4□ T5*□

(Note: - Study time point T0 = at discharge, T1 = 1 month, T2 = 3 month, T3* = 6month (lab test), T4 = 9 month, T5 = 12 month (lab test))

Name of the interviewer: ______________________________

Date of Interview: __________________

Time of Start of Interview: ______________________ (am/ pm)

Patient’s name (optional) __________________________

Study I .D. No (Unique ID given to each participant) ___________________

Patient’s address: _____________________________________________________________

Medical Record Number: - ____________________

Telephone no Residence: - ____________________

Mobile no: - ____________________________

Primary Care giver name (optional):-_________________________

Mobile no of primary care giver:-_____________________

Alternate Mobile number of primary care giver: - _______________

Referral Site (Name of hospital from where the patient is identified):-_________________

**FORM A, SECTION 1A:- SOCIODEMOGRAPHIC PROFILE**

| **Q** | **Variable** | **Code** | **Skip** | **Response** | **Variable type** |
| --- | --- | --- | --- | --- | --- |
| 1a. | **Date of Enrolment** | DOE |  | __ __ (dd)/ __ __(mm)/ __ __(yy) | NA |
| 1b. | **Date of Birth** | DOB |  | __ __ (dd)/ __ __(mm)/ __ __(yy) | NA |
| 1c. | **Age (in years)** | Actual Response |  | __ __ | Discrete |
| 1d. | **Gender** | 1. Male  2. Female |  | _________ | Nominal |
| 1e. | **Education**  **(Complete years of education)** | 1. Illiterate  2. Primary Education (1 to5)  3. Secondary Education (6 to10)  4. Higher Secondary Education  (11 and12)  5. Above Intermediate Education  (13 and above) |  | __________ | Categorical |
| 1f. | **Marital Status** | 1. Single  2. Married  3. Divorced  4. Widowed |  | __________ | Categorical |
| 1g. | **Family Status** | 1. Joint family  2. Nuclear family |  | __________ | Nominal |
| 1h. | **Monthly family income** | Actual Response |  | __ __ __ __ __ __ __ PKR | Categorical |
| 1i. | **How many house-hold members**  **Are there in the house?** | Actual Response |  | __ __ | Discrete |
| 1j. | **Employment status** | 1. Employed  2. Unemployed  3. Retired  4. Housewife  5. Daily wage  6. Others (specify) | >>>> 1l  >>>> 1l  >>>> 1l  >>>> 1l  >>>> 1l |  | Categorical |

| 1k. | **Occupation** | Occup. |  | _________________ |  |
| --- | --- | --- | --- | --- | --- |
| 1l. | **Household Assets**   - Washing machine - Color TV      - Cable TV - LCD - Refrigerator      - Tape recorder - Microwave - Freezer - CD Player - Sewing machine      - Car - Personal Computer      - Bicycle - Motor bike - Mobile phone - Cooking ware - Property - Air conditioner/split - Laptop | Household Assets |  | __ __  __ __  __ __  __ __  __ __  __ __  __ __  __ __  __ __  __ __  __ __  __ __  __ __  __ __  __ __  __ __  __ __  __ __  __ __ |  |
| 1m. | **Model of vehicle (s)** | Name of the vehicle  With its model |  | _____________________ |  |
| 1n. | **Land Ownership (if any)** | 1. None  2. Less than 1 acre  3. Between 1 and 10  Acres  4. More than 10 acres |  |  |  |
| 1o. | **Length of Hospital Stay (days)** | Days |  | __ __ |  |

**SECTION 1B:- MEDICAL DETAILS**

| **Q.** | **Variable** | **Code** | **Skip** | **Response** |
| --- | --- | --- | --- | --- |
| 1b (i) | Which type of Stroke the patient had? (etiology underlying stroke) | 1. Ischemic Stroke 2. Hemorrhage Stroke (ICH) |  | ___________________ |
| 1b (ii) | Did the patient have any of the co-morbid? | 1. Hypertension 2. Diabetes 3. Depression 4. Atrial Fibrillation 5. Peripheral Arterial Disease 6. Coronary Artery Disease 7. Dyslipidemia 8. Others (specify) |  | __  __  __  __  __  __  __  __________________________ |
| 1b (iii) | Which of the following forms of tobacco do you currently consume?  ( you can tick more than one) | 1. None 2. Cigarette 3. Sheesha 4. Cigar 5. Pipe 6. Huqqa 7. Biri 8. Niswar 9. Gutka 10. Beetle leaf/ Pan with or without tobacco |  | __  __  __  __  __  __  __  __  __  __ |
| 1b (iv) | Which of the following scans patient has undergone? | 1. MRI Scan 2. CT-Scan 3. Carotid Artery Scan 4. Echocardiography 5. Electrocardiogram |  | __  __  __  __  __ |
| 1b (v) | Patient is discharged on what medications? (list of medications) | Actual Response |  |  |
| 1b (vi) | Was tissue plasminogen activator (rtpa) given to the patient at the time of admission? | 1. Yes 2. No |  | __ |
| 1b (vi) | What was the Modified Rankin Scale (MRS) Score of the patient at the time of discharge? | Actual Response |  | _ _ |
| 1b (vii) | What was the NIH Stroke Scale Score of the patient at the time of discharge? | Actual Response |  | _ _ |
| 1b (viii) | What was the Barthel Index of the patient at the time of discharge? | Actual Response |  | _ _ |

**FORM B: FOLLOW-UP INTERVIEW**

**Name of the Interviewer: -** _________________________________________

**Date of Interview: - _**_ __ (dd)/ __ __ (mm)/ __ __ (yy)

**Time of Interview: -** ____________________ (am/pm)

**Name of the Patient: -** ______________________________________________

**SECTION 1:- ASSESSMENT OF FUNCTIONAL STATUS OF THE STROKE PATIENT**

**PART 1A: - MODIFIED RANKIN SCALE**

**
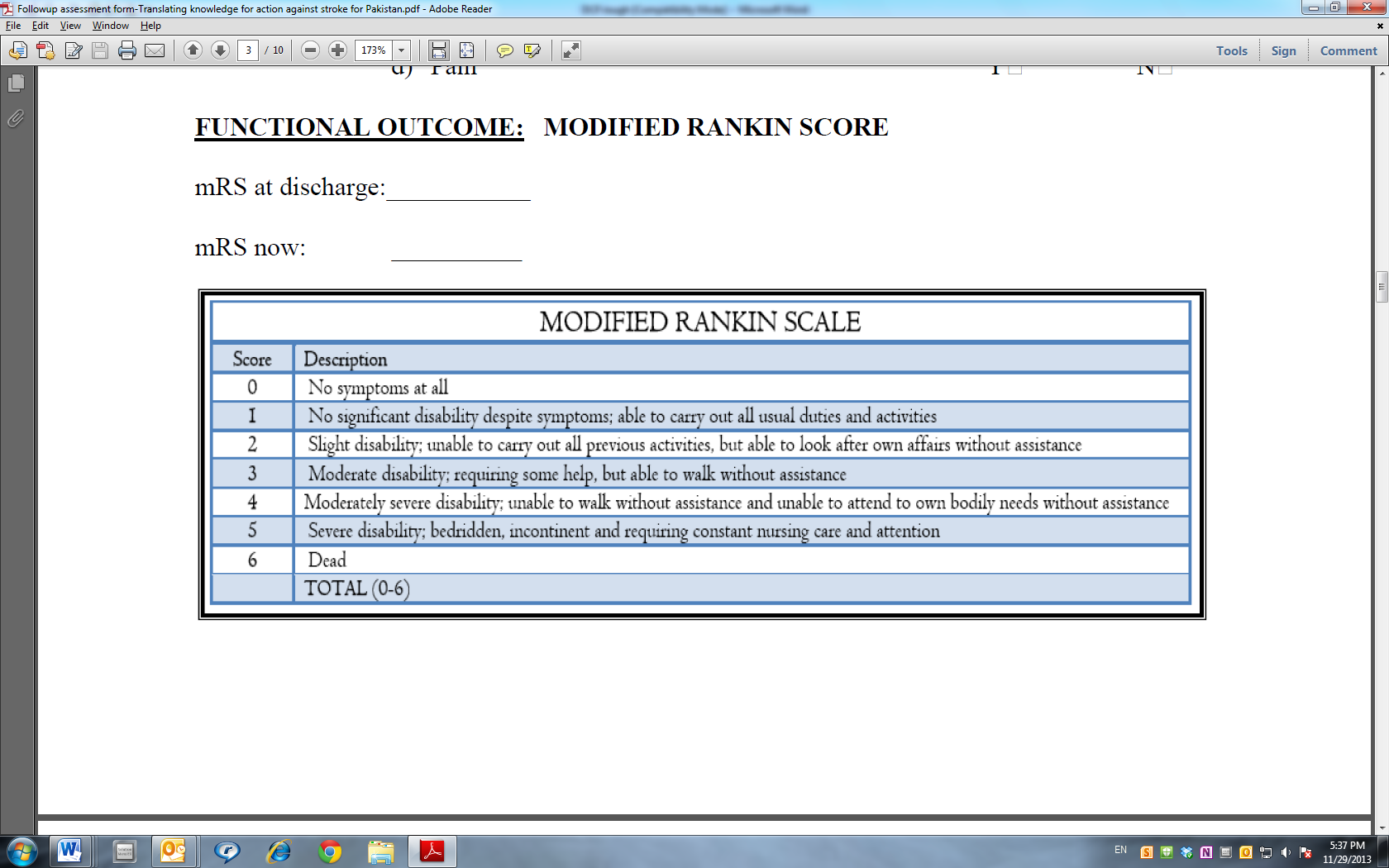
**

**PART 1B:- BARTHEL INDEX**

| **Activity** | **Score** |
| --- | --- |
| **Feeding**  0 = unable  5 = needs help cutting, spreading butter, etc., or requires modified diet  10 = independent | **_____** |
| **Bathing**  0 = dependent  5 = independent (or in shower) | **_____** |
| **Grooming**  0 = needs to help with personal care  5 = independent face/hair/teeth/shaving (implements provided) | **_____** |
| **Dressing**  0 = dependent  5 = needs help but can do about half unaided  10 = independent (including buttons, zips, laces, etc.) | **_____** |
| **Bowels**  0 = incontinent (or needs to be given enemas)  5 = occasional accident  10 = continent | **_____** |
| **Bladder**  0 = incontinent, or catheterized and unable to manage alone  5 = occasional accident  10 = continent | **_____** |
| **Toilet Use**  0 = dependent  5 = needs some help, but can do something alone  10 = independent (on and off, dressing, wiping) | **_____** |
| **Transfers (Bed to Chair and Back)**  0 = unable, no sitting balance  5 = major help (one or two people, physical), can sit  10 = minor help (verbal or physical)  15 = independent | **_____** |
| **Mobility (On Level Surfaces)**  0 = immobile or < 50 yards  5 = wheelchair independent, including corners, > 50 yards  10 = walks with help of one person (verbal or physical) > 50 yards  15 = independent (but may use any aid; for example, stick) > 50 yards | **_____** |
| **Stairs**  0 = unable  5 = needs help (verbal, physical, carrying aid)  10 = independent | **_____** |
| **Total Score (0-100)** | **_________** |

**PART 1 C. NIH STROKE SCALE**

| **Instructions** | **Scale Definition** | **Score** |
| --- | --- | --- |
| 1c (I a) **Level of Consciousness**: The investigator must choose a response if a full evaluation is prevented by such obstacles as an endo-tracheal tube, language barrier, orotracheal trauma/bandages. A 3 is scored only if the patient makes no movement (other than reflexive posturing) in response to noxious stimulation. | 0 = Alert; keenly responsive.  1 = Not alert; but arousable by minor stimulation to obey, answer, or respond.  2 = Not alert; requires repeated stimulation to attend, or is obtunded and requires strong or painful stimulation to make movements (not stereotyped).  3 = Responds only with reflex motor or autonomic effects or totally unresponsive, flaccid, and areflexic. | **________** |
| **1c (I b) LOC Questions:**  The patient is asked the month and his/her age. The answer must be correct - there is no partial credit for being close. Aphasic and stuporous patients who do not comprehend the questions will score 2. Patients unable to speak because of endotracheal intubation, orotracheal trauma, and severe dysarthria from any cause, language barrier, or any other problem not secondary to aphasia are given a 1. It is important that only the initial answer be graded and that the examiner not "help" the patient with verbal or non-verbal cues | 0 = Answers both questions correctly.    1 = Answers one question correctly.  2 = Answers neither question correctly. | **________** |
| **1c (I c) LOC Commands**:  The patient is asked to open and close the eyes and then to grip and release the non-paretic hand. Substitute another one step command if the hands cannot be used. Credit is given if an unequivocal attempt is made but not completed due to weakness. If the patient does not respond to command, the task should be demonstrated to him or her (pantomime), and the result scored (i.e., follows none, one or two commands). Patients with trauma, amputation, or other physical impediments should be given suitable one-step commands. Only the first attempt is scored. | 0 = Performs both tasks correctly.  1 = Performs one task correctly.  2 = Performs neither task correctly | **________** |
| **1 c (ii) Best Gaze:** Only horizontal eye movements will be tested. Voluntary or reflexive (oculocephalic) eye movements will be scored, but caloric testing is not done. If the patient has a conjugate deviation of the eyes that can be overcome by voluntary or reflexive activity, the score will be 1. If a patient has an isolated peripheral nerve paresis (CN III, IV or VI), score a 1. Gaze is testable in all aphasic patients. Patients with ocular trauma, bandages, pre-existing blindness, or other disorder of visual acuity or fields should be tested with reflexive movements, and a choice made by the investigator. Establishing eye contact and then moving about the patient from side to side will occasionally clarify the presence of partial gaze palsy. | 0 = Normal.  1 = Partial gaze palsy; gaze is abnormal in one or both eyes, but forced deviation and total gaze paresis is not present.  2 = Forced deviation, or total gaze paresis not overcome by the oculocephalic maneuver. | **________** |
| **1 c (iii) Visual:** Visual fields (upper and lower quadrants) are tested by confrontation, using finger counting or visual threat, as appropriate. Patients may be encouraged, but if they look at the side of the moving fingers appropriately, this can be scored as normal. If there is unilateral blindness or enucleation, visual fields in the remaining eye are scored. Score 1 only if a clear-cut asymmetry, including quadrantanopia, is found. If patient is blind from any cause, score 3. Double simultaneous stimulation is performed at this point. If there is extinction, patient receives a 1, and the results are used to respond to item 11. | 0 = No visual loss.  1 = Partial hemianopia.  2 =Complete hemianopia.  3 = Bilateral hemianopia (blind including cortical blindness). | **________** |
| **1c (iv) Facial Palsy:**  Facial Palsy: Ask – or use pantomime to encourage – the patient to show teeth or raise eyebrows and close eyes. Score symmetry of grimace in response to noxious stimuli in the poorly responsive or non-comprehending patient. If facial trauma/bandages, orotracheal tube, tape or other physical barriers obscure the face, these should be removed to the extent possible. | 0 = Normal symmetrical movements.  1 = Minor paralysis (flattened naso labial fold, asymmetry on smiling).  2 = Partial paralysis (total or near-total paralysis of lower face).  3 = Complete paralysis of one or both sides (absence of facial movement in the upper and lower face). | **________** |
| **1c (v) Motor Arm:**  The limb is placed in the appropriate position: extend the arms (palms down) 90 degrees (if sitting) or 45 degrees (if supine). Drift is scored if the arm falls before 10 seconds. The aphasic patient is encouraged using urgency in the voice and pantomime, but not noxious stimulation. Each limb is tested in turn, beginning with the non-paretic arm. Only in the case of amputation or joint fusion at the shoulder, the examiner should record the score as untestable (UN), and clearly write the explanation for this choice. | 0 = No drift; limb holds 90 (or 45) degrees for full 10 seconds.  1 = Drift; limb holds 90 (or 45) degrees, but drifts down before full 10 seconds; does not hit bed or other support.  2 = Some effort against gravity; limb cannot get to or maintain (if cued) 90 (or 45) degrees, drifts down to bed, but has some effort against gravity.  3 = No effort against gravity; limb falls.  4 = No movement.  UN = Amputation or joint fusion, explain  V a. Left Arm Score  V b. Right Arm Score | **__________________**  **__________________**  **__________________**  **__________________** |
| 1c (vi) **Motor Leg:** The limb is placed in the appropriate position: hold the leg at 30 degrees (always tested supine). Drift is scored if the leg falls before 5 seconds. The aphasic patient is encouraged using urgency in the voice and pantomime, but not noxious stimulation. Each limb is tested in turn, beginning with the non-paretic leg. Only in the case of amputation or joint fusion at the hip, the examiner should record the score as untestable (UN), and clearly write the explanation for this choice. | 0 = No drift; leg holds 30-degree position for full 5 seconds.  1 = Drift; leg falls by the end of the 5-second period but does not hit bed.  2 = Some effort against gravity; leg falls to bed by 5 seconds, but has some effort against gravity.  3 = No effort against gravity; leg falls to bed immediately.  4 = No movement.  UN = Amputation or joint fusion, explain    VI a. Left Leg  VI b. Right Leg | **__________________**  **__________________**  **__________________**  **__________________** |
| **1c (vii) Limb Ataxia:**  This item is aimed at finding evidence of a unilateral cerebellar lesion. Test with eyes open. In case of visual defect, ensure testing is done in intact visual field. The finger-nose-finger  and heel-shin tests are performed on both sides, and ataxia is scored only if present out of proportion to weakness. Ataxia is absent in the patient who cannot understand or is paralyzed. Only in the case of amputation or joint fusion, the examiner should record the score as untestable (UN), and clearly write the explanation for this choice. In case of blindness, test by having the patient touch nose from extended arm position. | 0 = Absent.  1 = Present in one limb.  2 = Present in two limbs.  UN = Amputation or joint fusion, explain | **____________**  **__________________** |
| **1c (viii) Sensory:**  Sensation or grimace to pinprick when tested, or withdrawal from noxious stimulus in the obtunded or aphasic patient. Only sensory loss attributed to stroke is scored as abnormal and the examiner should test as many body areas (arms [not hands], legs, trunk, face) as needed to accurately check for hemi sensory loss. A score of 2, “severe or total sensory loss,” should only be given when a severe or total loss of sensation can be clearly demonstrated. Stuporous and aphasic patients will, therefore, probably score 1 or 0.  The patient with brainstem stroke who has bilateral loss of sensation is scored 2. If the patient does not respond and is quadriplegic, score 2. Patients in a coma (item 1a=3) are automatically given a 2 on this item. | 0 = Normal; no sensory loss.  1 =Mild-to-moderate sensory loss; patient feels pinprick is less sharp or is dull on the affected side; or there is a loss of superficial pain with pinprick, but patient is aware of being touched.  2 = Severe to total sensory loss; patient is not aware of being touched in the face, arm, and leg. | **________** |
| **1c (ix) Best Language:**  A great deal of information about comprehension will be obtained during the preceding sections of the examination. For this scale item, the patient is asked to describe what is happening in the attached picture, to name the items on the attached naming sheet and to read from the attached list of sentences. Comprehension is judged from responses here, as well as to all of the commands in the preceding general neurological exam. If visual loss interferes with the tests, ask the patient to identify objects placed in the hand, repeat, and produce speech. The intubated patient should be asked to write. The patient in a coma (item 1a=3) will automatically score 3 on this item. The examiner must choose a score for the patient with stupor or limited cooperation, but a score of 3 should be used only if the patient is mute and follows no one-step commands | 0 = No aphasia; normal.  1 = Mild-to-moderate aphasia; some obvious loss of fluency  or facility of comprehension, without significant limitation on ideas expressed or form of expression. Reduction of speech and/or comprehension, however, makes conversation about provided materials difficult or impossible. For example, in conversation about provided materials, examiner can identify picture or naming card content from patient’s response.    2 = Severe aphasia; all communication is through fragmentary expression; great need for inference, questioning, and guessing by the listener. Range of information that can be exchanged is limited; listener carries burden of communication. Examiner cannot identify materials provided from patient response.  3 = Mute, global aphasia; no usable speech or auditory comprehension. | **________** |
| **1c (x) Dysarthria:**  If patient is thought to be normal, an adequate sample of speech must be obtained by asking patient to read or repeat words from the attached list. If the patient has severe aphasia, the clarity of articulation of spontaneous speech can be rated. Only if the patient is intubated or has other physical barriers to producing speech, the examiner should record the score as untestable (UN), and clearly write an explanation for this choice. Do not tell the patient why he or she is being tested. | 0 = Normal.  1 = Mild-to-moderate dysarthria; patient slurs at least some words and, at worst, can be understood with some difficulty.  2 = Severe dysarthria; patient's speech is so slurred as to be unintelligible in the absence of or out of proportion to any dysphasia, or is mute/anarthric.  UN = Intubated or other physical barrier,  explain | **________**  **__________________** |
| **1c (xi) Extinction and Inattention (formerly Neglect):**  Sufficient information to identify neglect maybe obtained during the prior testing. If the patient has a severe visual loss preventing visual double simultaneous stimulation, and the cutaneous stimuli are normal, the score is normal. If the patient has aphasia but does appear to attend to both sides, the score is normal. The presence of visual spatial neglect or anosagnosia may also be taken as evidence of abnormality. Since the abnormality is scored only if present, the item is never untestable. | 0 = No abnormality.  1 = Visual, tactile, auditory, spatial, or personal inattention or extinction to bilateral simultaneous stimulation in one of the sensory modalities.  2 = Profound hemi-inattention or extinction to more than one modality; does not recognize own hand or orients to only one side of space. | **________** |

**Total Score: - _______________**

**SECTION 2A:- QUALITY OF LIFE OF STROKE SURVIVORS BY STROKE SPECIFIC QUALITY OF LIFE SCALE**

| Total help - Couldn't do it at all - Strongly agree | 1 |
| --- | --- |
| A lot of help - A lot of trouble - Moderately agree | 2 |
| Some help - Some trouble - Neither agree nor disagree | 3 |
| A little help - A little trouble - Moderately disagree | 4 |
| No help needed - No trouble at all - Strongly disagree | 5 |

| **S.No** |  | **Score** |
| --- | --- | --- |
| **Energy** | |  |
| 1. | I felt tired most of the time |  |
| 2. | I had to stop and rest during the day |  |
| 3. | I was too tired to do what I wanted to do. |  |
|  |  |  |
| **Family Role** | |  |
|  | I didn't join in activities just for fun with my family |  |
|  | I felt I was a burden to my family |  |
|  | My physical condition interfered with my personal life |  |
| **Language** | |  |
|  | Did you have trouble speaking? For example, get stuck, stutter, stammer, or slur your words? |  |
|  | Did you have trouble speaking clearly enough to use the telephone? |  |
|  | Did other people have trouble in understanding what you said? |  |
|  | Did you have trouble finding the word you wanted to say? |  |
|  | Did you have to repeat yourself so others could understand you? |  |
| **Mobility** | |  |
|  | Did you have trouble walking? |  |
|  | Did you lose your balance when bending over to or reaching for something? |  |
|  | Did you have trouble climbing stairs? |  |
|  | Did you have to stop and rest more than you would like when walking or using a wheelchair? |  |
|  | Did you have trouble with standing? |  |
|  | Did you have trouble getting out of a chair? |  |
| **Mood** | |  |
|  | I was discouraged about my future. |  |
|  | I wasn't interested in other people or activities |  |
|  | I felt withdrawn from other people. |  |
|  | I had little confidence in myself |  |
|  | I was not interested in food |  |
| **Personality** | |  |
|  | I was irritable |  |
|  | I was inpatient with others |  |
|  | My personality has changed |  |
| **Self-Care** | |  |
|  | Did you need help preparing food? |  |
|  | Did you need help eating? For example, cutting food or preparing food |  |
|  | Did you need help getting dressed? For example, putting on socks or shoes, buttoning buttons, or zipping? |  |
|  | Did you need help taking a bath or a shower? |  |
|  | Did you need help to use the toilet? |  |
| **Social roles** | |  |
|  | I didn't go out as often as I would like. |  |
|  | I did my hobbies and recreation for shorter periods of time than I would like |  |
|  | I didn’t see as many of my friends as I would like. |  |
|  | My physical condition interfered with my social life |  |
| **Thinking** | |  |
|  | It was hard for me to concentrate |  |
|  | I had trouble remembering things. |  |
|  | I had to write things down to remember them |  |
| **Upper Extremity Function** | |  |
|  | Did you have trouble writing or typing? |  |
|  | Did you have trouble putting on socks? |  |
|  | Did you have trouble buttoning buttons? |  |
|  | Did you have trouble zipping a zipper? |  |
|  | Did you have trouble opening a jar? |  |
| **Vision** | |  |
|  | Did you have trouble seeing the television well enough to enjoy a show |  |
|  | Did you have trouble reaching things because of poor eyesight? |  |
|  | Did you have trouble seeing things off to one side? |  |
| **Work/Productivity** | |  |
|  | Did you have trouble doing daily work around the house? |  |
|  | Did you have trouble finishing jobs that you started? |  |
|  | Did you have trouble doing the work you used to do? |  |

**2B. MORISKY MEDICATION ADHERENCE QUESTIONNAIRE**

Individuals have identified several issues regarding their medication-taking behavior and we are interested in your experiences. There is no right or wrong answer. Please answer each question based on your personal experience with your stroke medication.

|  | **Question** | **Options** | **Skip** | **Answer** |
| --- | --- | --- | --- | --- |
| A.32.1 | Do you sometimes forget to take your stroke pills? | 0.Yes  1. No |  |  |
| A.32.2 | People sometimes miss taking their medications for reasons other than forgetting. Thinking over the past two weeks, were there any days when you did not take your stroke medicine | 0.Yes  1. No |  |  |
| A.32.3 | Have you ever cut back or stopped taking your medication without telling your doctor, because you felt worse when you took it? | 0.Yes  1.No |  |  |
| A.32.4 | When you travel or leave home, do you sometimes forget to bring along your stroke medication? | 0.Yes  1.No |  |  |
| A.32.5 | Did you take all your stroke medicines yesterday? | 0.Yes  1. No |  |  |
| A.32.6 | When you feel like your health is better, do you sometimes stop taking your medicine? | 0.Yes  1. No |  |  |
| A.32.7 | Taking medication every day is a real inconvenience for some people. Do you ever feel hassled about sticking to your stroke treatment plan? | 0.Yes  1.No |  |  |
| A.32.8 | How often do you have difficulty remembering to take all your medications? | 4.Never/Rarely  3.Once in a while  2. Sometimes  1.Usually  0. All the time |  |  |

Total Score ______________

**2**C. Post-Stroke Complications Requiring Medical Attention (Composite End-point)

| **Question** | **Description** | **Code** | **Skip** | **Response**  **(No. of Complications)** | **Variable type** |
| --- | --- | --- | --- | --- | --- |
| 4 a. **Any of the stroke related complications requiring**  **Readmission to hospital?** | • Recurrent stroke or  Transient ischemic attack  • Aspiration pneumonia  • Deep venous thrombosis  • Urinary tract infections  • Hemiplegic shoulder  • Neuropathic pain  • Falls  • Dysphagia  • Bedsores | 1. Yes  2. No | >>>> 4b | ________________ | Nominal |
| 4 b. **Any of the stroke**  **complications that require**  **medical attention but not**  **Readmission to hospital?** | • Recurrent stroke or  Transient ischemic attack  • Aspiration pneumonia  • Deep venous thrombosis  • Urinary tract infections  • Hemiplegic shoulder  • Neuropathic pain  • Falls  • Dysphagia  • Bedsores | 1. Yes  2. No | >>>> 4c | _________________ | Nominal |
| 4 c. **Stroke related mortality** | Assessed through Verbal  Autopsy Scale | 1.Probable cause of  death  2. Possible cause of  death  3. Mortality not  related to stroke | >>>>Sec D. | __________________ | Nominal |


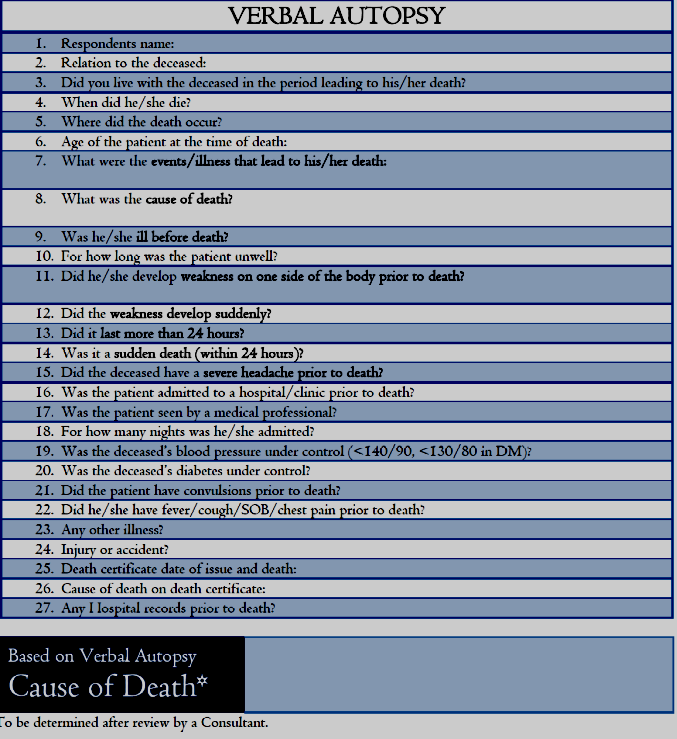


**2D. BLOOD PHYSIOLOGY (Only at discharge, 6 month and 12 month)**

| **BLOOD TEST** | **Time** | | |
| --- | --- | --- | --- |
|  | **T0 (at discharge)** | **T3 (6^th^ month)** | **T4 (12^th^ month)** |
| **HbA1C** |  |  |  |
| **BP** |  |  |  |
| **FBS** |  |  |  |
| **Cholesterol** |  |  |  |

**2E. QUESTIONNAIRE FOR STROKE RELATED KNOWLEDGE ASSESSMENT**

| **Question** | **Code** | **Skip** | **Response** |
| --- | --- | --- | --- |
| 2 (i) **What causes the stroke**  **To happen?** | NA |  |  |
| 2 (ii) **What are the risk**  **Factors for stroke?** | NA |  |  |
| 2 (iii) **Describe precautionary**  **measures to avoid risk factors**  **For Stroke?** | NA |  |  |
| 2 (iv) **What should you do**  **immediately if you find that**  **your patient’s blood glucose level is**  **Less than 100mg/dl?** | NA |  |  |
| 2 (v) **What should you do**  **immediately if you find that**  **your patient’s systolic blood pressure**  **Is >140mmHg?** | NA |  |  |
| 2 (vi) **What are the warning**  **Signs of stroke?** | NA |  |  |
| 2 (vii) **What should you**  **immediately do if your patient**  **experience any of the warning**  **Signs of stroke?** | NA |  |  |
| 2 (viii) **What measures shall**  **you take, to make your**  **environment at home, safe for**  **The stroke patient?** | NA |  |  |
| 2 (ix) **How can your patient regain**  **the movement of body**  **Parts?** | NA |  |  |
| 2 (x) **What should you do to**  **avoid constipation in the**  **Stroke patients?** | NA |  |  |

| 2 (xi) **How should oral hygiene**  **care be performed to avoid**  **Pneumonia?** | NA |  |  |
| --- | --- | --- | --- |
| 2 (xii) **What points shall be**  **kept in mind while preparing**  **Food for the stroke patients?** | NA |  |  |
| 2 (xiii) **How do you ensure safety of the stroke patients during exercises?** | NA |  |  |
| 2 (xiv) **What type of food shall**  **be given to patients who has**  **Had stroke one month back?** | NA |  |  |
| 2 (xv) **What type of food shall**  **be given to the patient who has**  **gained some control over**  **Chewing and swallowing?** | NA |  |  |

**SECTION 3:- ECONOMIC QUESTIONS RELATED TO STROKE EXPENDITURE** (COST ASSESSMENT) AFTER DISCHARGE FROM HOSPITAL

| **S.NO** | **QUESTIONS** | **Yes** | **No** | **Comments if any?** |
| --- | --- | --- | --- | --- |
| 1 | Does the patient have medical coverage from any organization? |  |  |  |
| 2 | Did the patient after suffering from stroke received any alternative medicine treatment? |  | If no, go to Q.3. |  |
| 2.1 | If yes, then what is the expenditure of this informal care or alternative medicine treatment for last one month? |  |  |  |
| 3 | Does the patient receive rehabilitation service on regular basis?  Means the rehabilitation services specified by the primary physician or the rehabilitation expert. |  | If no, go to Q.4. |  |
| 3.1 | If yes, then from which place whether a rehabilitation center? |  |  |  |
| 3.2 | Or does the patient receive rehabilitation services at home by trained personnel? |  |  |  |
| 3.3 | Can you please tell the amount of money spend on rehabilitation services for last one month? |  |  |  |
| 3.4 | At what time the patient started receiving the rehabilitation services after occurrence of stroke?(please specify in number of days) |  |  |  |
| 4 | Please mention medications of the stroke patient? |  |  |  |
| 4.1 | Can you please tell the amount of money spend on medicines of the stroke patient for last one month? |  |  |  |
| 5 | Can you please tell the amount of money spend on travelling for stroke patient for receiving health services? |  |  |  |
| 6 | Can you please tell the amount of money spend on consultation cost for stroke patient with the doctors for last one month? |  |  |  |
| 7 | Can you please tell roughly the amount of money spend on the lab procedures for stroke patient if any for one month? |  |  |  |
| 8 | Can you please tell the amount of money spend for any special food preparations for stroke patient for the last one month? |  |  |  |
| 9 | Can you please tell the amount of money that was spent on re-hospitalization when the patient suffered from any of the stroke complications or secondary stroke? |  |  |  |
| 10 | As the primary care giver can you please tell your time that was lost because of providing care to the stroke patient which otherwise would have been utilized for performing some sort of leisure activity by you? |  |  |  |
| 11 | Did you need to leave job because of providing care to the stroke patients? |  |  |  |
| 12. | Did your working hours changed because of providing care to stroke patients? |  |  |  |

**Form C (Intervention Arm Patient)**

**Name of the Interviewer: -** _________________________________________

**Date of Interview: -_**_ __ (dd)/ __ __ (mm)/ __ __ (yy)

**Time of Interview: -** ____________________ (am/pm)

**Name of the Patient: -** _____________________________________________

**2F. VIDEO ASSESMENT**

**Comprehensive assessment of video content:**

Number of video:

Title of video set: __________________

1^st^ □ 2^nd^ □ 3^rd^ □ 4^th^ □

- Which video did you find most difficult to understand?

________________________________________________________________________

________________________________________________________________________

________________________________________________________________________

________________________________________________________________________

________________________________________________________________________

- Which video did you find easy to understand?

________________________________________________________________________

________________________________________________________________________

________________________________________________________________________

________________________________________________________________________

________________________________________________________________________

- Which video was most helpful to you?

________________________________________________________________________

________________________________________________________________________

________________________________________________________________________

________________________________________________________________________

________________________________________________________________________

- Which video were least helpful for you?

_______________________________________________________________________

_______________________________________________________________________

________________________________________________________________________

________________________________________________________________________

________________________________________________________________________

- What aspects of video did you like most? Why?

________________________________________________________________________

________________________________________________________________________

________________________________________________________________________

________________________________________________________________________

________________________________________________________________________

- What aspects of video did you dislike most? Why?

________________________________________________________________________

_______________________________________________________________________

________________________________________________________________________

________________________________________________________________________

_____________________________________________________________________________­­­­­­­­­­­­­­­­­­­­­­­­­­­­___________________________________________________________________

2G. PATIENT SATISFACTION WITH MOVIES FOR STROKE TRIAL

|  | **QUESTION** | **Options** | **Answer** |
| --- | --- | --- | --- |
| G.2.1 | Using movies for stroke is a good way to learn the rehabilitation skills | 1.Yes  0.No |  |
| G.2.2 | I enjoyed movies for stroke program | 1.Yes  0.No |  |
| G.2.3 | I was able to understand all the movies for stroke | 1.Yes  0.No |  |
| G.2.4 | I recognize risk factors for stroke well through movies | 1.Yes  0.No |  |
| G.2.5 | I got to know what has happened to me through movies for stroke | 1.Yes  0.No |  |
| G.2.6 | I got to know which diet is best for me immediately after stroke through movies | 1.Yes  0.No |  |
| G.2.7 | I would like to watch these movies again for better understanding and comprehension | 1.Yes  0.No |  |
| G.2.8 | I would recommend this movie program to a friend  or family member | 1.Yes  0.No |  |
| G.2.9 | I was motivated by these sets of movies | 1.Yes  0.No |  |
| G.2.10 | The movies on medicines helped me remember to take my medicines on time and be regular with it | 1.Yes  0.No |  |
| G.2.11 | I couldn’t understand some set of movies | 0.Yes  1.No |  |
| G.2.12 | The language of the videos was difficult to understand | 0.Yes  1.No |  |
| G.2.13 | Do you think the videos contain too much information for you to grasp? | 0.Yes  1.No |  |
|  |  | Total Score | ________ |

H. ACCEPTABILITY OF mHealth INNOVATIONS

| S.No | Questions | 1. YES/ 0. NO |
| --- | --- | --- |
| H.3.1 | You learned about your health more quickly and easily because of watching *Videos for stroke* |  |
| H.3.2 | You think patients with diseases other than stroke should also use Videos for health education |  |
| H.3.3 | You enjoyed learning about your health watching *Videos for stroke* |  |
| H.3.4 | People can tell that you know more about your health since you have watched videos *for stroke* |  |
| H.3.5 | You would have no difficulty in telling friends what *different set of videos for stroke*  are like |  |
| H.3.6 | *Videos for stroke* helped you learn about your drugs and your disease |  |
| H.3.7 | You had no difficulty in watching all set *videos for stroke* and they were not boring |  |

**Total Score: ______________**

***Date of the Interview:-***

***_ _ /_ _ /_ _***

***(dd/mm/yy)***

***Name of the person who filled the form****:-*

*___________________ (Response)*

***Signature of the person who filled the form:-***

*___________________*

***Name of the person who edited the form****:-*

*___________________ (Response)*

***Signature of the person who edited the form:-***

*___________________*

***Name of the person who entered the form****:-*

*___________________ (Response)*

***Signature of the person who entered the form***

*___________________*
