# Supplementary material for: Translating knowledge for action against stroke – using 5-minute videos for stroke survivors and caregivers to improve post-stroke outcomes: study protocol for a randomized controlled trial (Movies4Stroke)
Source: Trials. 2016 Jan 27;17:52. doi: 10.1186/s13063-016-1175-x (PMC4728820; doi:10.1186/s13063-016-1175-x)
Supplement: Additional file 2: — Data Collection Form (DCF) for Caregiver. (DOC 209 kb) [file 13063_2016_1175_MOESM2_ESM.doc]

**Appendix**

**ELIGIBILITY FORM**

**IDENTIFICATION DATA:**

1. Serial Number for eligibility :- _______________________
2. Medical Record No. :- _________________________
3. Name of the patient:- _______________________________
4. Mobile Number of the patient :- _______________________
5. Address of the patient:- _________________________________________________
6. Name of the Care-giver:- ____________________________
7. Mobile Number of the Care-giver:- ____________________
8. Address of the Care-giver:- ______________________________________________

**PATIENTS FILE REVIEW:**

Date of Medical File Review: ___________________

| **S.No** | **Eligibility Criteria** | **Yes** | **No** |
| --- | --- | --- | --- |
| 1 | Age >18 yrs. |  |  |
| 2 | Admitted with first ever stroke (acute stroke) |  |  |
| 3 | Able to understand Urdu (languages of the video) |  |  |
| 4 | Have a stable surrogate care-giver |  |  |
| 5 | Modified Rankin Score < 4 (mild to moderate stroke) |  |  |
| 6 | No intention to travel for next 12 months |  |  |
| 7 | Permanent resident of Karachi |  |  |
| 8 | No history of short term memory loss or any visual or hearing loss in the patient and caregiver that interferes with the understanding of the videos |  |  |
| 9 | No history of serious concurrent medical illnesses, like cancer, renal failure , chronic liver disease or acute liver disease in the past 6 months |  |  |
| 10 | Those patients not to be included having non- atherosclerotic vascular stroke or stroke from rare causes for e.g. gunshot to neck, carotid dissection and post CABG ,etc. |  |  |
| 11 | No history of any unique co morbid that interfere with patient’s medication compliance e.g. a liver failure patient that can’t take statins or a surgery is planned so aspirin needs to be stopped. |  |  |

All criteria should be answered in YES to be eligible for participation in the study (both patient along with caregiver).

**This patient is eligible to participate in the Video-Based Intervention for stroke patients and caregivers**

**YES NO**

If the patient and care-giver are eligible but refuses to participate please state the reason for

Non-participation: ______________________________________

**MOVIES FOR STROKE PATIENTS/CAREGIVER DYAD**

**PHONE INFRA STRUCTURE DATA**

1. **BRAND NAME OF THE CELL PHONE: _________________________**
2. **TYPE OF OPERATING SYSTEM IN THE CELL PHONE:**

**i) ANDROID**

**ii) WINDOWS**

**iii) IOS**

1. **DOES ANY ONE AT HOME HAVE AN ANDROID CELLULAR PHONE?**
2. **YES**
3. **NO**
4. **DO YOU HAVE A DESKTOP PC AT HOME?**
5. **YES**
6. **NO**

**Data Collection Form For Care-givers**

Study time point: T0□ T1□ T2□ T3*□ T4□ T5*□

(Note: - Study time point T0 = at discharge, T1 = 1 month, T2 = 3 month, T3* = 6month (lab test), T4 = 9 month, T5 = 12 month (lab test))

Name of the interviewer: ______________________________

Date of Interview: __________________

Time of Start of Interview: ______________________ (am/ pm)

Patient’s name (optional) __________________________

Study I .D. No (Unique ID given to each participant) ___________________

Patient’s address: _____________________________________________________________

Medical Record Number: - ____________________

Telephone no Residence: - ____________________

Mobile no: - ____________________________

Primary Care giver name (optional):-_________________________

Mobile no of primary care giver:-_____________________

Alternate Mobile number of primary care giver: - _______________

Referral Site (Name of hospital from where the patient is identified):-_________________

**FORM A, SECTION 1A:- SOCIODEMOGRAPHIC PROFILE**

| **Q** | **Variable** | **Code** | **Skip** | **Response** | **Variable type** |
| --- | --- | --- | --- | --- | --- |
| 1a. | **Date of Enrolment** | DOE |  | __ __ (dd)/ __ __(mm)/ __ __(yy) | NA |
| 1b. | **Date of Birth** | DOB |  | __ __ (dd)/ __ __(mm)/ __ __(yy) | NA |
| 1c. | **Age (in years)** | Actual Response |  | __ __ | Discrete |
| 1d. | **Gender** | 1. Male  2. Female |  | _________ | Nominal |
| 1e. | **Education**  **(Complete years of education)** | 1. Illiterate  2. Primary Education (1 to5)  3. Secondary Education (6 to10)  4. Higher Secondary Education  (11 and12)  5. Above Intermediate Education  (13 and above) |  | __________ | Categorical |
| 1f. | **Marital Status** | 1. Single  2. Married  3. Divorced  4. Widowed |  | __________ | Categorical |
| 1g. | **Family Status** | 1. Joint family  2. Nuclear family |  | __________ | Nominal |
| 1h. | **Monthly family income** | Actual Response |  | __ __ __ __ __ __ __ PKR | Categorical |
| 1i. | **How many house-hold members**  **Are there in the house?** | Actual Response |  | __ __ | Discrete |
| 1j. | **Employment status** | 1. Employed  2. Unemployed  3. Retired  4. Housewife  5. Daily wage  6. Others (specify) | >>>> 1l  >>>> 1l  >>>> 1l  >>>> 1l  >>>> 1l |  | Categorical |

| 1k. | **Occupation** | Occup. |  | _________________ |  |
| --- | --- | --- | --- | --- | --- |
| 1l. | **Household Assets**   - Washing machine - Color TV      - Cable TV - LCD - Refrigerator      - Tape recorder - Microwave - Freezer - CD Player - Sewing machine      - Car - Personal Computer      - Bicycle - Motor bike - Mobile phone - Cooking ware - Property - Air conditioner/split - Laptop | Household Assets |  | __ __  __ __  __ __  __ __  __ __  __ __  __ __  __ __  __ __  __ __  __ __  __ __  __ __  __ __  __ __  __ __  __ __  __ __  __ __ |  |
| 1m. | **Model of vehicle (s)** | Name of the vehicle  With its model |  | _____________________ |  |
| 1n. | **Land Ownership (if any)** | 1. None  2. Less than 1 acre  3. Between 1 and 10  Acres  4. More than 10 acres |  |  |  |
| 1o. | **Length of Hospital Stay (days)** | Days |  | __ __ |  |

**FORM B: FOLLOW-UP INTERVIEW**

**Name of the Interviewer: -** _________________________________________

**Date of Interview: - _**_ __ (dd)/ __ __ (mm)/ __ __ (yy)

**Time of Interview: -** ____________________ (am/pm)

**Name of the Care-giver: -** ______________________________________________

**SECTION 1: CAREGIVER BURDEN SCALE**

| Zarit et al. (1980), *Gerontologist, 20(6), 649‐55* | |  |  |  |  |  |  |
| --- | --- | --- | --- | --- | --- | --- | --- |
| Instructions: Read each statement and rate it on a scale from | 0 (never) to 4 (nearly always) | | | |  |  |  |
| In general, how often do you feel: | Never | | |  | Nearly Always | | |
| There is not enough time for yourself |  | 0 | 1 | 2 | 3 | 4 |  |
| Overtaxed with responsibilities |  | 0 | 1 | 2 | 3 | 4 |  |
| Like you’ve lost control over your life |  | 0 | 1 | 2 | 3 | 4 |  |
| In regard to the relative for whom you are caring, how often do you feel: | |  |  |  |  |  |  |
| Uncertain about what to do for your relative |  | 0 | 1 | 2 | 3 | 4 |  |
| Like you should do more for your relative |  | 0 | 1 | 2 | 3 | 4 |  |
| Like you could do a better job of caring |  | 0 | 1 | 2 | 3 | 4 |  |
|  | |  |  |  |  |  |  |
| When you are with the relative for whom you are caring, how often do you feel: | |  |  |  |  |  |  |
|  |  |  |  |  |  |  |  |
| A sense of strain |  | 0 | 1 | 2 | 3 | 4 |  |
| Anger |  | 0 | 1 | 2 | 3 | 4 |  |
| Embarrassment |  | 0 | 1 | 2 | 3 | 4 |  |
| Uncomfortable about having friends over |  | 0 | 1 | 2 | 3 | 4 |  |
| How often do you feel that your relationship with the relative for whom | |  |  |  |  |  |  |
| you’re caring negatively impacts: |  |  |  |  |  |  |  |
|  |  |  |  |  |  |  |  |
| Your social life |  | 0 | 1 | 2 | 3 | 4 |  |
| Other relationships with family and friends |  | 0 | 1 | 2 | 3 | 4 |  |
| Your health |  | 0 | 1 | 2 | 3 | 4 |  |
| Your privacy |  | 0 | 1 | 2 | 3 | 4 |  |
| How often do you: |  |  |  |  |  |  |  |
| Feel you receive excessive help requests |  | 0 | 1 | 2 | 3 | 4 |  |
| Feel all the responsibility falls on one caregiver |  | 0 | 1 | 2 | 3 | 4 |  |
| Fear the future regarding your relative |  | 0 | 1 | 2 | 3 | 4 |  |
|  | |  |  |  |  |  |  |
| Fear not having enough money to care for your relative | | 0 | 1 | 2 | 3 | 4 |  |
| Fear not being able to continue caring for your relative |  | 0 | 1 | 2 | 3 | 4 |  |
| Wish to leave the care of your relative to someone else |  | 0 | 1 | 2 | 3 | 4 |  |
| How much does your spouse/loved one depend on you as the caregiver? | | 0 | 1 | 2 | 3 | 4 |  |

Please rate your overall level of burden in caring for your spouse/relative:

1. No burden at all (1) Mild Burden (2) Moderate Burden (3) Severe Burden (4) Extreme Burden

**Total Score: -** ______________________________

**Interpretation:**

1. No or minimal burden: 0 to 20
2. Mild to moderate burden: 21 to 40
3. Moderate to severe burden: 41‐60
4. Severe burden: 61 to 88

**SECTION 2A. QUESTIONNAIRE FOR STROKE RELATED KNOWLEDGE ASSESSMENT (CARE-GIVERS)**

| **Question** | **Code** | **Skip** | **Response** |
| --- | --- | --- | --- |
| 2 (i) **What causes the stroke**  **To happen?** | NA |  |  |
| 2 (ii) **What are the risk**  **Factors for stroke?** | NA |  |  |
| 2 (iii) **Describe precautionary**  **measures to avoid risk factors**  **For Stroke?** | NA |  |  |
| 2 (iv) **What should you do**  **immediately if you find that**  **your patient’s blood glucose level is**  **Less than 100mg/dl?** | NA |  |  |
| 2 (v) **What should you do**  **immediately if you find that**  **your patient’s systolic blood pressure**  **Is >140mmHg?** | NA |  |  |
| 2 (vi) **What are the warning**  **Signs of stroke?** | NA |  |  |
| 2 (vii) **What should you**  **immediately do if your patient**  **experience any of the warning**  **Signs of stroke?** | NA |  |  |
| 2 (viii) **What measures shall**  **you take, to make your**  **environment at home, safe for**  **The stroke patient?** | NA |  |  |
| 2 (ix) **How can your patient regain**  **the movement of body**  **Parts?** | NA |  |  |
| 2 (x) **What should you do to**  **avoid constipation in the**  **Stroke patients?** | NA |  |  |

| 2 (xi) **How should oral hygiene**  **care be performed to avoid**  **Pneumonia?** | NA |  |  |
| --- | --- | --- | --- |
| 2 (xii) **What points shall be**  **kept in mind while preparing**  **Food for the stroke patients?** | NA |  |  |
| 2 (xiii) **How do you ensure safety of the stroke patients during exercises?** | NA |  |  |
| 2 (xiv) **What type of food shall**  **be given to patients who has**  **Had stroke one month back?** | NA |  |  |
| 2 (xv) **What type of food shall**  **be given to the patient who has**  **gained some control over**  **Chewing and swallowing?** | NA |  |  |

**SECTION 3:- ECONOMIC QUESTIONS RELATED TO STROKE EXPENDITURE** (COST ASSESSMENT) AFTER DISCHARGE FROM HOSPITAL

| **S.NO** | **QUESTIONS** | **Yes** | **No** | **Comments if any?** |
| --- | --- | --- | --- | --- |
| 1 | Does the patient have medical coverage from any organization? |  |  |  |
| 2 | Did the patient after suffering from stroke received any alternative medicine treatment? |  | If no, go to Q.3. |  |
| 2.1 | If yes, then what is the expenditure of this informal care or alternative medicine treatment for last one month? |  |  |  |
| 3 | Does the patient receive rehabilitation service on regular basis?  Means the rehabilitation services specified by the primary physician or the rehabilitation expert. |  | If no, go to Q.4. |  |
| 3.1 | If yes, then from which place whether a rehabilitation center? |  |  |  |
| 3.2 | Or does the patient receive rehabilitation services at home by trained personnel? |  |  |  |
| 3.3 | Can you please tell the amount of money spend on rehabilitation services for last one month? |  |  |  |
| 3.4 | At what time the patient started receiving the rehabilitation services after occurrence of stroke?(please specify in number of days) |  |  |  |
| 4 | Please mention medications of the stroke patient? |  |  |  |
| 4.1 | Can you please tell the amount of money spend on medicines of the stroke patient for last one month? |  |  |  |
| 5 | Can you please tell the amount of money spend on travelling for stroke patient for receiving health services? |  |  |  |
| 6 | Can you please tell the amount of money spend on consultation cost for stroke patient with the doctors for last one month? |  |  |  |
| 7 | Can you please tell roughly the amount of money spend on the lab procedures for stroke patient if any for one month? |  |  |  |
| 8 | Can you please tell the amount of money spend for any special food preparations for stroke patient for the last one month? |  |  |  |
| 9 | Can you please tell the amount of money that was spent on re-hospitalization when the patient suffered from any of the stroke complications or secondary stroke? |  |  |  |
| 10 | As the primary care giver can you please tell your time that was lost because of providing care to the stroke patient which otherwise would have been utilized for performing some sort of leisure activity by you? |  |  |  |
| 11 | Did you need to leave job because of providing care to the stroke patients? |  |  |  |
| 12. | Did your working hours changed because of providing care to stroke patients? |  |  |  |

**Form C (For Intervention Arm Only)**

**Name of the Interviewer: -** _________________________________________

**Date of Interview: -**  __ __ (dd)/ __ __ (mm)/ __ __ (yy)

**Time of Interview: -** ____________________ (am/pm)

**Name of the Care-giver: -** _____________________________________________

**2F. VIDEO ASSESMENT**

**Comprehensive assessment of video content:**

Number of video:

Title of video set: __________________

1^st^ □ 2^nd^ □ 3^rd^ □ 4^th^ □

- Which video did you find most difficult to understand?

________________________________________________________________________

________________________________________________________________________

________________________________________________________________________

________________________________________________________________________

________________________________________________________________________

- Which video did you find easy to understand?

________________________________________________________________________

________________________________________________________________________

________________________________________________________________________

________________________________________________________________________

________________________________________________________________________

- Which video was most helpful to you?

________________________________________________________________________

________________________________________________________________________

________________________________________________________________________

________________________________________________________________________

________________________________________________________________________

- Which video were least helpful for you?

_______________________________________________________________________

_______________________________________________________________________

________________________________________________________________________

________________________________________________________________________

________________________________________________________________________

- What aspects of video did you like most? Why?

________________________________________________________________________

________________________________________________________________________

________________________________________________________________________

________________________________________________________________________

________________________________________________________________________

- What aspects of video did you dislike most? Why?

________________________________________________________________________

_______________________________________________________________________

________________________________________________________________________

________________________________________________________________________

_____________________________________________________________________________­­­­­­­­­­­­­­­­­­­­­­­­­­­­___________________________________________________________________

2G. PATIENT SATISFACTION WITH MOVIES FOR STROKE TRIAL

|  | **QUESTION** | **Options** | **Answer** |
| --- | --- | --- | --- |
| G.2.1 | Using movies for stroke is a good way to learn the rehabilitation skills | 1.Yes  0.No |  |
| G.2.2 | I enjoyed movies for stroke program | 1.Yes  0.No |  |
| G.2.3 | I was able to understand all the movies for stroke | 1.Yes  0.No |  |
| G.2.4 | I recognize risk factors for stroke well through movies | 1.Yes  0.No |  |
| G.2.5 | I got to know what has happened to me through movies for stroke | 1.Yes  0.No |  |
| G.2.6 | I got to know which diet is best for me immediately after stroke through movies | 1.Yes  0.No |  |
| G.2.7 | I would like to watch these movies again for better understanding and comprehension | 1.Yes  0.No |  |
| G.2.8 | I would recommend this movie program to a friend  or family member | 1.Yes  0.No |  |
| G.2.9 | I was motivated by these sets of movies | 1.Yes  0.No |  |
| G.2.10 | The movies on medicines helped me remember to take my medicines on time and be regular with it | 1.Yes  0.No |  |
| G.2.11 | I couldn’t understand some set of movies | 0.Yes  1.No |  |
| G.2.12 | The language of the videos was difficult to understand | 0.Yes  1.No |  |
| G.2.13 | Do you think the videos contain too much information for you to grasp? | 0.Yes  1.No |  |
|  |  | Total Score | _________ |

H. ACCEPTABILITY OF mHealth INNOVATIONS

| S.No | Questions | 1. YES/ 0. NO |
| --- | --- | --- |
| H.3.1 | You learned about your health more quickly and easily because of watching *Videos for stroke* |  |
| H.3.2 | You think patients with diseases other than stroke should also use Videos for health education |  |
| H.3.3 | You enjoyed learning about your health watching *Videos for stroke* |  |
| H.3.4 | People can tell that you know more about your health since you have watched videos *for stroke* |  |
| H.3.5 | You would have no difficulty in telling friends what *different set of videos for stroke*  are like |  |
| H.3.6 | *Videos for stroke* helped you learn about your drugs and your disease |  |
| H.3.7 | You had no difficulty in watching all set *videos for stroke* and they were not boring |  |

**Total Score: ______________**

***Date of the Interview:-***

***_ _ /_ _ /_ _***

***(dd/mm/yy)***

***Name of the person who filled the form****:-*

*___________________ (Response)*

***Signature of the person who filled the form:-***

*___________________*

***Name of the person who edited the form****:-*

*___________________ (Response)*

***Signature of the person who edited the form:-***

*___________________*

***Name of the person who entered the form****:-*

*___________________ (Response)*

***Signature of the person who entered the form***

*___________________*
