# Supplementary material for: Translating knowledge for action against stroke – using 5-minute videos for stroke survivors and caregivers to improve post-stroke outcomes: study protocol for a randomized controlled trial (Movies4Stroke)
Source: Trials. 2016 Jan 27;17:52. doi: 10.1186/s13063-016-1175-x (PMC4728820; doi:10.1186/s13063-016-1175-x)
Supplement: Additional file 3: — Participant timeline. (DOCX 48 kb) [file 13063_2016_1175_MOESM3_ESM.docx]

**Translating knowledge for action against stroke - Using 5 minute videos for numeracy and literacy challenged stroke survivors and caregivers to improve outcomes**

**Screening for the eligibility criteria**

**(Patients and their caregivers)**

Prior to

Enrollment

**Randomize at Individual Level**

**T0**

***Assessment:- Assessment:-***

**HbA1C, BP, RBS, Cholesterol (Patient ) HbA1C, BP, RBS, Cholesterol (Patient)**

**Socio-Demographic Profile Socio-Demographic Profile**

**Medical Details (Patient) Medical Details (Patient)**

Pretest

At the time of

Discharge, 1^st^ set

of videos

2^nd^ set of videos

***Assessment: - Assessment:-***

**Functional Status (Stroke Patient) Functional Status (Stroke Patient) Quality of Life(Stroke Patient) Quality of Life (Stroke Patient)**

**Morisky Medication Adherence Scale (Patient) Morisky Medication Adherence Scale (Patient)**

**Post-Stroke Complications (Patient) Post Stroke Complications (Patient)**

**Knowledge Assessment (Patient & Caregiver) Knowledge Assessment (Patient & Caregiver)**

**Video Assessment (Patient& Care-giver) Cost Assessment (Patient & Caregiver)**

**Patient Satisfaction with mHealth Care-giver Burden Scale**

**Cost Assessment (Patient & Caregiver)**

**Care-giver Burden Scale**

**T1**

Time Point2

At 1^st^month

Follow-up

In clinic

Study Visit 2

3^rd^set of videos

***Assessment: Assessment:-***

**Functional Status (Stroke Patient) Functional Status (Stroke Patient) Quality of Life(Stroke Patient) Quality of Life (Stroke Patient)**

**Morisky Medication Adherence Scale (Patient) Morisky Medication Adherence Scale (Patient)**

**Post-Stroke Complications (Patient) Post Stroke Complications (Patient)**

**Knowledge Assessment (Patient & Caregiver) Knowledge Assessment (Patient & Caregiver)**

**Video Assessment (Patient& Care-giver) Cost Assessment (Patient & Caregiver)**

**Patient Satisfaction with mHealth Care-giver Burden Scale**

**Cost Assessment (Patient & Caregiver)**

**Care-giver Burden Scale**

**T2**

Time Point 3

At 3^rd^ month

Study Visit 3

4^th^ set of videos

***Assessment: Assessment:-***

**Functional Status (Stroke Patient) Functional Status (Stroke Patient) Quality of Life (Stroke Patient) Quality of Life (Stroke Patient)**

**Morisky Medication Adherence Scale (Patient) Morisky Medication Adherence Scale (Patient)**

**Post-Stroke Complications (Patient) Post Stroke Complications (Patient)**

**Knowledge Assessment (Patient & Caregiver) Knowledge Assessment (Patient & Caregiver)**

**Video Assessment (Patient & Care-giver) Cost Assessment (Patient & Caregiver)**

**Patient Satisfaction with mHealth HbA1C, BP, RBS, Cholesterol (Patient)**

**Cost Assessment (Patient & Caregiver) Care-giver Burden Scale**

**HbA1C, BP, RBS, Cholesterol (Patient)**

**Care-giver Burden Scale**

**T3**

Time Point 4

At 6^th^ month

Study Visit 4

**T4**

***Assessment: Assessment:-***

**Functional Status (Stroke Patient) Functional Status (Stroke Patient) Quality of Life(Stroke Patient) Quality of Life (Stroke Patient)**

**Morisky Medication Adherence Scale (Patient) Morisky Medication Adherence Scale (Patient)**

**Post-Stroke Complications (Patient) Post Stroke Complications (Patient)**

**Knowledge Assessment (Patient & Caregiver) Knowledge Assessment (Patient & Caregiver)**

**Video Assessment (Patient & Care-giver) Cost Assessment (Patient & Caregiver)**

**Patient Satisfaction with mHealth Care-giver Burden Scale**

**Cost Assessment (Patient & Caregiver)**

**Care-giver Burden Scale**

Time point 5

At 9 months

Study visit 5

***Assessment: -Assessment:-***

**Functional Status (Stroke Patient) Functional Status (Stroke Patient) Quality of Life(Stroke Patient) Quality of Life (Stroke Patient)**

**Morisky Medication Adherence Scale (Patient) Morisky Medication Adherence Scale (Patient)**

**Post-Stroke Complications (Patient) Post Stroke Complications (Patient)**

**Knowledge Assessment (Patient & Caregiver) Knowledge Assessment (Patient & Caregiver)**

**Video Assessment (Patient & Care-giver) Cost Assessment (Patient & Caregiver)**

**Patient Satisfaction with mHealth HbA1C, BP, RBS, Cholesterol (Patient)**

**Cost Assessment (Patient & Caregiver) Care-giver Burden Scale**

**HbA1C, BP, RBS, Cholesterol (Patient)**

**Care-giver Burden Scale**

**T5**

Time point 6

At 12 months

Study visit 6

Final

Assessment

**Primary end points**

**Effects of HBA1C, BP, Cholesterol, RBS**

**Medication adherence, QOL**

**Secondary end points: Burden of caregiver, Post stroke complications, Knowledge Assessment (patients/caregivers)**

**Primary end points**

**Effects of HBA1C, BP, Cholesterol, RBS**

**Medication adherence, QOL**

**Secondary end points: Burden of caregiver, Post stroke complications, Knowledge Assessment (patients/caregivers)**

**Secondary end point: Burden of caregiver**
