# Supplementary material for: Translating knowledge for action against stroke – using 5-minute videos for stroke survivors and caregivers to improve post-stroke outcomes: study protocol for a randomized controlled trial (Movies4Stroke)
Source: Trials. 2016 Jan 27;17:52. doi: 10.1186/s13063-016-1175-x (PMC4728820; doi:10.1186/s13063-016-1175-x)
Supplement: Additional file 4: — Thematic Intervention Chart. (DOCX 17 kb) [file 13063_2016_1175_MOESM4_ESM.docx]

**Video Thematic Intervention Chart**

| **Timing** | **Theme Information for Caregivers and Skills** | **Theme Emergency Preparedness** | **Theme Adherence to Medications** | **Theme**  **Stroke Prevention** |
| --- | --- | --- | --- | --- |
| Admission | ***** |  |  |  |
| At the time of Discharge |  | * |  |  |
| 1^st^ Month after discharge |  |  | * |  |
| 3^rd^ Month after discharge |  |  |  | * |

**Video Titles and Topics:**

| **Video Theme** |  |
| --- | --- |
| **Theme Information for Caregivers and Skills ( Admission )** | **Theme Emergency Preparedness (Prior to Discharge)** |
| I have had a stroke – what happens now?  Rehab at home  Swallowing difficulty , recognition and exercises  PEG and NGT feeding | Stroke recognition as an emergency and calling an ambulance  Emergency phone numbers video in phones  Heart attacks  Hypoglycemia  Seizure first aid  CPR – Basic |
| **Theme Medication Adherence and Understanding (First month after Stroke )** | **Theme Stroke Prevention (Stable Three Months after stroke )** |
| Medications for stroke are permanent  Why do I take this medication? What is the good? What should I watch out for? – Aspirin Warfarin , lipid lowering, blood pressure meds | Eating more fruit and vegetables  Primary prevention of stroke- exercise ,physical activity  Recognition of depression , atypical depression CBT –  Low salt diet  Smoking – how we quit  Hypertension – how to measure it, what are the cut offs, |
