# Supplementary material for: Translating knowledge for action against stroke – using 5-minute videos for stroke survivors and caregivers to improve post-stroke outcomes: study protocol for a randomized controlled trial (Movies4Stroke)
Source: Trials. 2016 Jan 27;17:52. doi: 10.1186/s13063-016-1175-x (PMC4728820; doi:10.1186/s13063-016-1175-x)
Supplement: Additional file 5: — Consent Form. (DOCX 20 kb) [file 13063_2016_1175_MOESM5_ESM.docx]

**Informed Consent**

**Project Title:**

Translating knowledge for action against stroke - Using 5 minute videos for numeracy and literacy challenged stroke survivors and caregivers to improve outcomes

| **Project Information** | |
| --- | --- |
| **ERC Ref No :** 2890-Med-ERC-14 | Sponsor: University Research Council, Aga Khan University (URC 132001 MED) |
| **Principal Investigator**: Dr. Ayeesha Kamal | **Organization:** Aga Khan University |
| **Location:** Stroke Unit and Ward, Aga Khan University | **Phone:** 9221-3486-4559 |
| **Other Investigators**: Dr. Adeel Khoja, Dr. Mariam, Dr. Asma Ahmed, Dr. Saleem Sayani, Dr. Sarah Saleem, Iqbal Azam, Abdul Muqeet and Dr. Nabila Soomro | **Organization:** Aga Khan University, AKDN, eHealth Resource Centre, Endocrinology, Psychology, Nursing and Rehabilitation. |
| **Location:** Aga Khan University, Hospital, Karachi | **Phone:** 9221-3486-4559 |

**PURPOSE OF THIS RESEARCH STUDY**

You are being asked to participate in a study on stroke patients in which we will examine the effect of video based health education sessions upon the adherence, QOL, and knowledge of the stroke patients and their caregivers.

**PROCEDURES**

You are being asked to participate in a study on stroke patients in which we will examine the effect of video based health education sessions upon the adherence, QOL, and knowledge of the stroke patients and their caregivers.

If you get a chance to be selected in an intervention group, you and your caregiver will be provided comprehensive education on stroke including its risk factors, treatment regimen, self-management and skills to deal with stroke disabilities. This education will be conducted via a movie session. The movie will be installed in your mobile phone so that you may watch them at home as well. There will be four movie sessions throughout the study. At each follow up visit you and your caregiver (who is enrolled in the study) will be asked some questions related to your health and knowledge about the stroke.

If you are selected in the control group, you and your caregiver will be educated by your nurse, doctors and other members of the health care team, for stroke management regimen. At each follow-up visit, you and your caregiver will be asked some questions related to your health and knowledge about the stroke.

**POSSIBLE RISKS OR DISCOMFORT**

Apparently the study does not impose any risk or discomfort to any patient or their caregiver. You will have to come for the follow-up visits at the scheduled time, set at the time of enrollment in the study.

**POSSIBLE BENEFITS**

You will receive educational movies free of cost that will improve your knowledge and skills to manage your illness at home and prevent from potential complications.

**FINANCIAL CONSIDERATIONS**

The cost of the blood test will be funded by the study, regardless of your assignment, whether you get video education or not.

**AVAILABLE TREATMENT ALTERNATIVES**

The alternative source of health education is the conventional form of health education provided by the physicians and nurses which are inadequate to develop behavioural changes among the patients.

**AVAILABLE MEDICAL TREATMENT FOR ADVERSE EXPERIENCES**

It is not expected that you will incur a medical treatment as a result of Visual education. However since you are our study participant, we will assist in directing you to your correct care provider should an unrelated medical issue arise during the course of the study. Additionally, your participation in this study will not affect the care and follow up that you will continue to receive in the stroke clinic.

**CONFIDENTIALITY**

Your identity in this study will be kept confidential and the results of the study will not disclose your identity. Your details will be given unique identity numbers or codes to maintain confidentiality. However any records or data obtained as a result of your participation in this study may be inspected by the sponsor or the AKU ERC members.

**TERMINATION OF RESEARCH STUDY**

The results of the study will be communicated to you. You are free to choose whether or not to participate in this study. There will be no penalty or loss of benefits to which you are otherwise entitled in this hospital. In case of your discontinuation of participation please notify Stroke Team at 0332-3668923 of your decision at the following provided telephone number so that your participation can be terminated.

**AVAILABLE SOURCES OF INFORMATION**

- Any further questions you have about this study will be answered by the Principal Investigator

**Name:** Dr Ayeesha Kamal

**Phone Number:** 0332-3668923

- Any questions you may have about your rights as a research subject will be answered by

**Name:** Dr Ayeesha Kamal

**Phone Number:** 0332-3668923

**AUTHORIZATION**

I have read and understood this consent form, and I volunteer to participate in this research study. I understand that I will receive a copy of this form. I voluntarily choose to participate, but I understand that my consent does not take away any legal rights in the case of negligence or other legal fault of anyone who is involved in this study.

**Name of participant: ______________**

**Signature of participant:______________**Date: _ _ / _ _ / _ _

(dd / mm / yy)

**Signature of Principal Investigator:______________**

Date : _ _ / _ _ / _ _

(dd / mm / yy)

**Signature of Person Obtaining Consent: ______________**

Date: _ _ / _ _ / _ _

(dd / mm / yy)
