# Supplementary material for: Translating knowledge for action against stroke – using 5-minute videos for stroke survivors and caregivers to improve post-stroke outcomes: study protocol for a randomized controlled trial (Movies4Stroke)
Source: Trials. 2016 Jan 27;17:52. doi: 10.1186/s13063-016-1175-x (PMC4728820; doi:10.1186/s13063-016-1175-x)
Supplement: Additional file 7: — Review of previous studies. (DOCX 31 kb) [file 13063_2016_1175_MOESM7_ESM.docx]

| **Article Name**  **Additional File 10: Stroke Caregivers Support Studies Review** | **Population** | **Major Outcome Variable** | **Important**  **Findings** | **Knowledge Gap** |
| --- | --- | --- | --- | --- |
| **Training carers of stroke patients: randomized controlled trial**  Kalra L, Evans A, Perez I, Melbourn A, Patel A, Knapp M, et al.  BMJ: British Medical Journal. 2004; 328(7448):1099. (1) | Stroke Survivors and their informal Caregivers | - Patient Mortality, Hospitalization and Disability - Quality of Life and Mood Outcome of Caregiver - Cost-Effectiveness | - A significant difference was observed with respect to the quality of life and mood outcome among Care-givers - Reduction in Cost | - This trial at least points out the value of intense education and promoting health literacy after stroke, although it does not test the power of digital media to achieve this |
| **A structured training programme for caregivers of inpatients after stroke (TRACS): a cluster randomized controlled trial and cost-effectiveness analysis**  Forster A, Dickerson J, Young J, Patel A, Kalra L, Nixon J, et al.  The Lancet. 2013. (2) | Stroke Survivors and their informal Caregivers | - Extended Activities of Daily Living - Cost-Effectiveness - Care-giver Burden Scale | - No differences between the London Stroke Carers Training Course and Usual Care on any of the assessed outcomes | - This trial failed to utilize unique media or mobile health resources and recommended more follow-up after discharge |
| **Stress in caregivers of aphasic stroke patients: a randomized controlled trial**.  Draper B, Bowring G, Thompson C, Van Heyst J, Conroy P, Thompson J Clinical Rehabilitation 2007; 21:122–30. (3) | Stroke Survivors and their informal Caregivers | - Impact of a psycho-education programme on caregivers’ burden and stress and communication between the caregiver and aphasic stroke patient. | - Stroke caregiver support, education and training programmes have short-term effects on caregiver stress levels. | - This trial proposes ongoing involvement to maintain effect of intervention. |
| **Telephone intervention with family caregivers of stroke survivors after rehabilitation.**  Grant JS, Elliot TR, Weaver M, Bartolucci AA, Newman Giger J. Stroke 2002; 33: 2060–5. (4) | Stroke Survivors and their informal Caregivers | Impact of social problem-solving telephone partnerships on primary family caregiver outcomes. | - Caregivers of intervention group had better problem-solving skills; greater preparedness; less depression; and significant improvement in other measures | - Healthcare professionals are further advised to develop effective intervention programs that will assist caregivers to effectively manage caregiving problems. |
| **Telephone group intervention for older stroke caregivers.** Hartke RJ, King RB. Topics in Stroke Rehabilitation 2003; 9(4):65–81. (5) | Stroke Survivors and their informal Caregivers | Evaluating the effectiveness of telephone groups for older, spousal caregivers of stroke survivors | - Treatment participants showed decreased stress over time and a significant increase in competence | - Assessment of costs failed to include the initial investment in developing the training intervention. |
| **The impact of a nurse-led support and education programme for spouses of stroke patients: randomized controlled trial.** Larson J, Franzen-Dahlin A, Billing E, Von Arbin M, Murray V, Wredling R. Journal of Clinical Nursing 2005; 14:995–1003. (6) | Stroke Survivors and their informal Caregivers | Determining the impact of a nurse-led support and education programme for improving the spouses’ perceived general quality of life, life situation, general well-being and health state. | - No significant differences were found, between intervention and control groups, over time | - Further development of the support and education programme is needed, including empowerment approach and implementation of coping strategies. |
| **Family support for stroke: a randomised controlled trial.**  Mant J, Carter J, Wade DT, Winner S. Lancet 2000; 356 (9232):808–13. (7) | Stroke Survivors and their informal Caregivers | Assessing the impact of family support on stroke patients and their carers. | - Family support significantly increased social activities and improved quality of life for carers, with no significant effects on patients. | - Proposed studies in different settings to provide effective services to improve quality of life of informal caregivers. |
| **The effect of a Web-based stroke intervention on carer’s well-being and survivors’ use of health care services.**  Pierce LL, Steiner VL, Khuder SA, Govoni AL, Horn LJ. Disability and Rehabilitation 2009; 31(20):1676–84. (8) | Stroke Survivors and their informal Caregivers | Predicted that those survivors whose carers participated in Caring-Web would use fewer healthcare services. | - No statistical differences were found between the groups in carers’ well-being or in the number of provider visits for survivors. | - Proposed studies in different settings to find an association between intervention and the carers’ well-being. |
| **The effects of a support group intervention on the burden of primary family caregivers of stroke patients.** Taehan Kanho Hakhoe Chi 2007; 37(5): 693–702. (9) | Stroke Survivors and their informal Caregivers | Developing and evaluating the effects of a support group intervention on the burden of primary family caregivers of [stroke](http://europepmc.org/abstract/med/17804936/?whatizit_url=http://europepmc.org/search/?page=1&query=%22stroke%22) patients | - The experimental group had a significantly lower total burden score and sub-scales of emotional, time-dependent and developmental burden scores than the control group | - Suggested that a support group intervention can be utilized as an effective nursing program. |

**References:**

1. Kalra L, Evans A, Perez I, Melbourn A, Patel A, Knapp M, et al. Training carers of stroke patients: randomised controlled trial. Bmj. 2004;328(7448):1099.

2. Forster A, Dickerson J, Young J, Patel A, Kalra L, Nixon J, et al. A structured training programme for caregivers of inpatients after stroke (TRACS): a cluster randomised controlled trial and cost-effectiveness analysis. The Lancet. 2014;382(9910):2069-76.

3. Draper B, Bowring G, Thompson C, Van Heyst J, Conroy P, Thompson J. Stress in caregivers of aphasic stroke patients: a randomized controlled trial. Clinical Rehabilitation. 2007;21(2):122-30.

4. Grant JS, Elliott TR, Weaver M, Bartolucci AA, Giger JN. Telephone intervention with family caregivers of stroke survivors after rehabilitation. Stroke. 2002;33(8):2060-5.

5. Hartke RJ, King RB. Telephone group intervention for older stroke caregivers. Topics in Stroke Rehabilitation. 2003;9(4):65-81.

6. Larson J, Franzén‐Dahlin Å, Billing E, Arbin M, Murray V, Wredling R. The impact of a nurse‐led support and education programme for spouses of stroke patients: a randomized controlled trial. Journal of clinical nursing. 2005;14(8):995-1003.

7. Mant J, Carter J, Wade DT, Winner S. Family support for stroke: a randomised controlled trial. The Lancet. 2000;356(9232):808-13.

8. Pierce LL, Steiner VL, Khuder SA, Govoni AL, Horn LJ. The effect of a Web-based stroke intervention on carers' well-being and survivors' use of healthcare services. Disability and rehabilitation. 2009;31(20):1676-84.

9. Yoo EK, Jeon S, Yang JE. [The effects of a support group intervention on the burden of primary family caregivers of stroke patients]. Taehan Kanho Hakhoe Chi. 2007;37(5):693-702.
